# Convergence of Multidimensional Sensing: A Review of AI-Enhanced Space-Division Multiplexing in Optical Fiber Sensors

**DOI:** 10.3390/s26072044

**Published:** 2026-03-25

**Authors:** Rabiu Imam Sabitu, Amin Malekmohammadi

**Affiliations:** 1 Photonic Communication and Signal Processing Research Group, Department of Physics, Aliko Dangote University of Science and Technology, Kano 711011, Nigeria; 2 Department of Computer and Electrical Engineering, California State University, Bakersfield, CA 93311, USA; aminmalek_m@ieee.org

**Keywords:** optical fiber sensors, space-division multiplexing, multi-core fiber, few-mode fiber, artificial intelligence, machine learning, distributed sensing, structural health monitoring

## Abstract

The growing demand for high-fidelity, multi-parameter, distributed sensing in critical domains such as structural health monitoring, oil and gas exploration, and secure perimeter surveillance is pushing traditional optical fiber sensors (OFS) to their performance limits. Although conventional multiplexing techniques such as time-division and wavelength-division multiplexing (TDM, WDM) have been commercially successful, they are rapidly approaching fundamental bottlenecks in sensor density, spatial resolution, and data capacity. This review argues that the synergistic convergence of space-division multiplexing (SDM) and artificial intelligence (AI) represents a paradigm shift, enabling a new generation of intelligent, high-dimensional sensing networks. We comprehensively survey the state of the art in SDM-based OFS, detailing the operating principles and applications of multi-core fibers (MCFs) for ultra-dense sensor arrays and 3D shape sensing, as well as few-mode fibers (FMFs) for mode-division multiplexing and enhanced multi-parameter discrimination. However, the unprecedented spatial parallelism provided by SDM introduces significant challenges, including inter-channel crosstalk, complex signal demultiplexing, and massive data volumes. This paper systematically explores how AI, particularly machine learning (ML) and deep learning (DL), is being leveraged not merely as a tool but as an indispensable core technology to mitigate these impairments. We critically analyze AI’s role in digital crosstalk suppression, intelligent mode demultiplexing, signal denoising, and solving complex inverse problems for parameter estimation. Furthermore, we highlight how this AI–SDM synergy enables capabilities beyond the reach of either technology alone, such as super-resolution sensing and predictive analytics. The discussion is extended to include the critical supporting pillars of this ecosystem, such as advanced interrogation techniques and the associated data management challenges. Finally, we provide a forward-looking perspective on the trajectory of the field, outlining a path toward cognitive sensing networks that are self-calibrating, adaptive, and capable of autonomous decision-making. This review is intended to serve as a foundational reference for researchers and engineers at the intersection of photonics and intelligent systems, illuminating the pathway toward tomorrow’s intelligent sensing infrastructure.

## 1. Introduction

The 21st-century infrastructural and industrial landscape is increasingly defined by the integration of sensing technologies, creating “smart” systems capable of self-monitoring and informed decision-making. At the heart of this transformation lies the demand for robust, dense, distributed sensing networks that can operate in harsh and expansive environments, from the depths of oil wells to the spans of long-span bridges and aerospace vehicles [1]. Among the various sensing technologies, optical fiber sensors (OFS) have emerged as a preeminent solution, offering a unique combination of immunity to electromagnetic interference (EMI), intrinsic safety, small size, and the capacity for multiplexing and distributed sensing over tens of kilometers [2,3].

**Figure 1:**
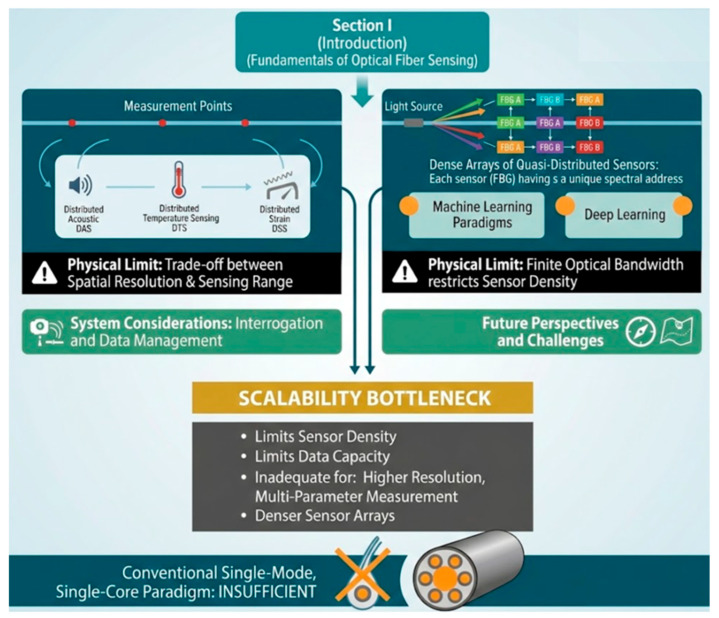
Optical Fiber Sensing: Capabilities and limits of classical multiplexing [14].

The capabilities of OFS have been largely unlocked through classical multiplexing techniques. Time-division multiplexing (TDM) forms the basis of distributed acoustic (DAS), temperature (DTS), and strain (DSS) sensing, allowing measurements at every point along a fiber [4,5]. Wavelength-division multiplexing (WDM) enables the deployment of dense arrays of quasi-distributed sensors, such as fiber Bragg gratings (FBGs), by assigning each sensor a unique spectral address [5]. Despite their commercial success, these approaches are confronting fundamental physical limits as illustrated in Figure 1. The trade-off between spatial resolution and sensing range in TDM, and the finite optical bandwidth in WDM, create a scalability bottleneck that restricts sensor density and data capacity [6]. As applications evolve to require higher spatial resolutions, simultaneous multi-parameter measurement, and denser sensor arrays, the conventional single-mode, single-core fiber model is proving inadequate.

To overcome this bottleneck, the field has begun to explore the spatial dimension of the fiber itself [7,8,9], drawing inspiration from optical communications. Space-division multiplexing (SDM) represents a paradigm shift, moving beyond a single spatial channel to employ specialized fibers [10]. Multi-core fibers (MCFs) incorporate multiple independent cores within a single cladding, multiplying sensing capacity by the number of cores and enabling applications like 3D shape sensing [11]. Few-mode fibers (FMFs) exploit distinct spatial modes as independent sensing channels, each with unique sensitivity to environmental perturbations, facilitating sophisticated multi-parameter discrimination from a single fiber [12].

The promise of SDM is profound, but it introduces significant complexities: inter-core and inter-mode crosstalk, challenging signal demultiplexing, and a dramatic increase in raw data volume. For example, a 32-core DAS system generates ~9.2 GB/hour, exceeding conventional processing capacity [13].

### 1.1. The AI Imperative: From Data Deluge to Cognitive Sensing

Coinciding with the rise in SDM is the transformative progress in artificial intelligence (AI). The intricate, high-dimensional data generated by SDM-based OFS presents a challenge ideally suited to AI techniques. AI is no longer a secondary tool but is rapidly becoming a core processing engine for OFS, demonstrating remarkable efficacy in denoising weak scattering signals [15], classifying vibration events [16], and solving complex inverse problems [17]. The application of AI to SDM-based OFS is a natural and necessary evolution, offering pathways to mitigate its inherent challenges. For example, using neural networks for digital crosstalk suppression [18] and to unlock new functionalities, such as super-resolution sensing and intelligent data fusion.

This review posits that the synergistic convergence of SDM and AI is forging a new frontier in optical fiber sensing. SDM provides the rich, multidimensional spatial canvas; AI supplies the computational intelligence to interpret it. This partnership is bidirectional: AI makes SDM-based OFS practical and powerful through crosstalk suppression and super-resolution sensing, while SDM-based OFS provides the complex, high-value data that drive advanced AI development.

### 1.2. Review Scope and Structure

**Figure 2:**
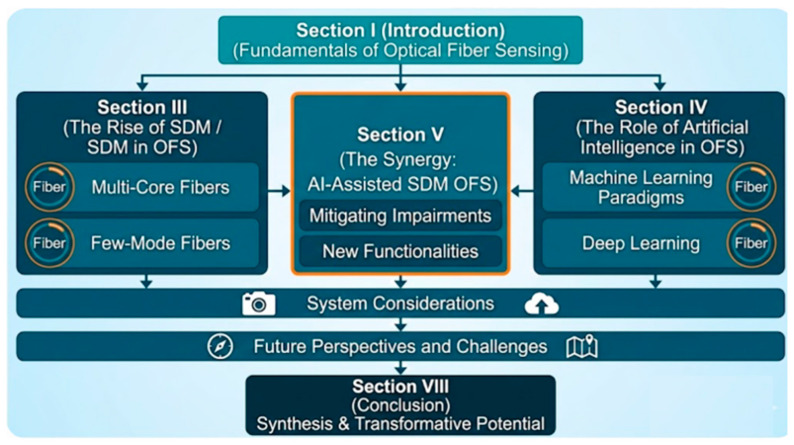
Conceptual map of the paper’s structure [19,20].

This paper reviews AI-enhanced SDM optical fiber sensing across eight sections as illustrated in Figure 2. Section 2 details the systematic methodology. Section 3 covers OFS fundamentals: waveguide theory, transduction mechanisms, and performance metrics. Section 4 presents SDM architectures—MCFs, FMFs, and bundled arrays—with microscopic images, addressing the data deluge and AI-SDM convergence. Section 5 delivers the core framework: AI indispensability, inverse problem formulation, problem-centric AI mapping (Table 4), quantitative benchmarking (Table 5), and ML paradigms (Table 6). Section 6 examines interrogation technologies, data management, edge/cloud AI, and standardization. Section 7 proposes a four-layer roadmap—efficiency, data economy, trust, and hardware integration—toward cognitive sensing networks. Section 8 concludes with key insights and future perspectives.

## 2. Methodology and Contributions

### 2.1. Methodology of This Review

This review employs a systematic and critical analysis methodology to synthesize the current state of the art at the intersection of SDM and AI in optical fiber sensing. The methodology ensures comprehensive coverage, objective evaluation, and identification of clear future pathways through four key stages:

1. Literature Identification and Collection: An extensive search was conducted across major scientific databases (IEEE Xplore, Scopus, Web of Science, OSA Publishing, MDPI) using structured keywords including “optical fiber sensor,” “space division multiplexing,” “multi-core fiber,” “few-mode fiber,” “artificial intelligence,” and “machine learning,” combined with Boolean operators.
2. Screening and Categorization: Identified literature was screened for relevance and categorized into a structured framework:
  - SDM Fundamentals: Design, fabrication, and primary sensing principles of MCFs and FMFs.
  - SDM Sensing Applications: Use of SDM fibers for shape sensing and multi-parameter discrimination.
  - AI in Conventional OFS: Foundational work applying AI/ML to standard single-core OFS.
  - AI-for-SDM Impairment Mitigation: Studies using AI to address crosstalk, demultiplexing, and signal recovery.
  - AI for Enhanced SDM Functionality: Research where AI extracts new information beyond conventional methods.
  - Interrogation and System Design: Critical interrogation hardware and system architectures.
3. Critical Analysis and Synthesis: Within each category, strengths, limitations, and key findings were critically evaluated. Synthesis focused on drawing connections across categories, identifying overarching trends, and highlighting knowledge gaps, supported by comparative tables.
4. Formulation of Future Perspectives: Based on this synthesis, evidence-based projections for future research directions were developed, including cognitive sensing networks extrapolated from current trends.

### 2.2. Key Contributions of This Review

**Figure 3:**
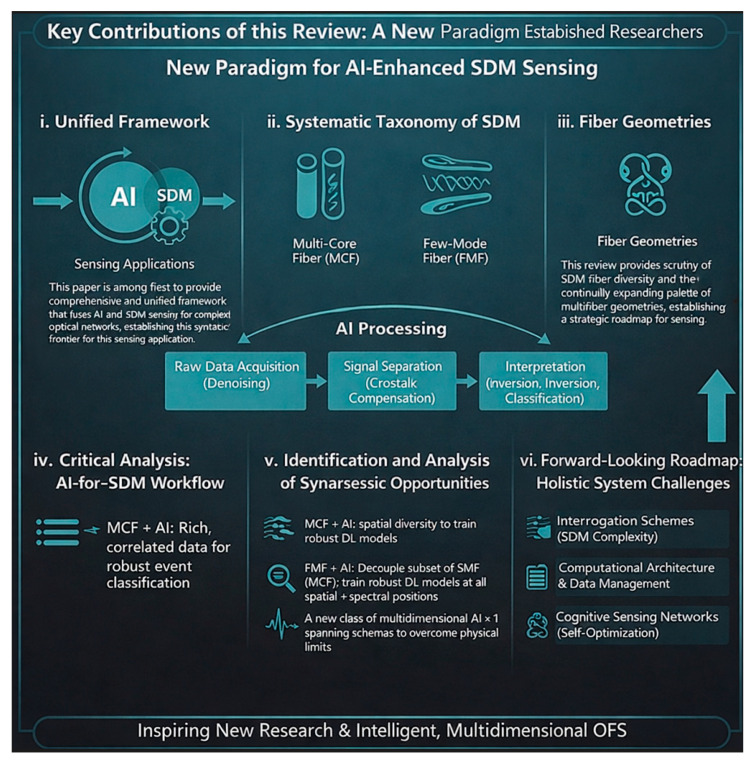
Proposed framework for AI-enhanced SDM sensing, showing a unified workflow from data acquisition to interpretation, key contributions, and future roadmaps for multi-core and few-mode fibers [21].

This review makes five key contributions:

1. First unified framework for AI-enhanced SDM sensing: Provides a comprehensive framework explicitly unifying SDM and AI for sensing applications, focusing on their synergistic interplay rather than treating them as separate topics (Figure 3).
2. Systematic taxonomy of SDM fiber geometries for sensing: Presents a structured classification of SDM fiber types (MCFs, FMFs, bundled fibers) tailored to the sensing community, detailing unique opportunities and challenges from parallelization to multi-parameter extraction.
3. Critical analysis of the AI-for-SDM workflow: Organizes the role of AI along the entire SDM-OFS signal chain, from raw data acquisition through signal separation to final interpretation—providing a practical guide for system designers.
4. Identification of synergistic opportunities: Identifies specific areas where SDM-AI combination creates value greater than the sum of parts, including MCF spatial diversity for robust AI training, AI decoupling of complex FMF modal responses, and super-resolution techniques overcoming physical sensing limits.
5. Forward-looking roadmap addressing holistic system challenges: Extends beyond the sensor fiber to encompass interrogation schemes, computational architectures, and data management strategies, culminating in a vision for self-optimizing “cognitive” sensing networks.

## 3. Fundamentals of Optical Fiber Sensing

### 3.1. Optical Fiber Sensing Principles: A Concise Overview

OFS have become indispensable for precision measurement in structural health monitoring, industrial process control, and environmental sensing. Their fundamental advantages include immunity to electromagnetic interference, compact form factor enabling minimally invasive integration, and inherent multiplexing capability. This supports kilometer-scale distributed sensing, derived directly from their dielectric waveguide nature and the interaction of guided light with external perturbations.

Waveguide Fundamentals: Light propagates through total internal reflection in a core (refractive index n_1_) surrounded by cladding (n_2_ < n_1_). Single-mode fibers (SMFs) guide only the fundamental mode, offering high precision but limited data capacity. Multimode fibers (MMFs) support multiple spatial modes, increasing light collection at the cost of modal dispersion that limits spatial resolution. Chromatic dispersion—wavelength-dependent group velocity—further constrains performance in both fiber types, particularly for distributed sensing where pulse broadening directly impacts resolution.

**Figure 4:**
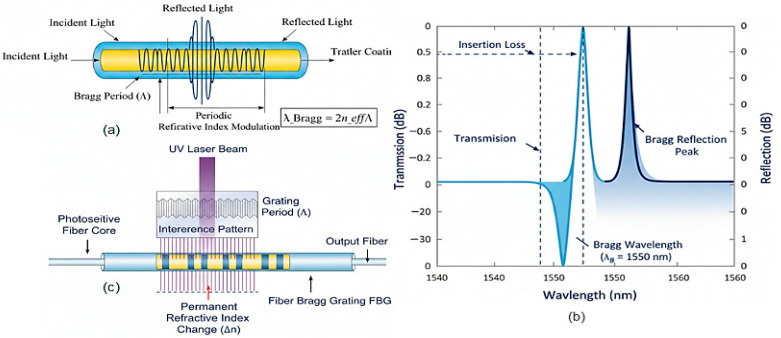
(**a**) Fiber Bragg grating structure with periodic refractive index modulation. (**b**) Reflection and transmission spectra of a typical FBG. (**c**) UV inscription process for FBG fabrication [25].

Physical Transduction Mechanisms: OFS convert environmental changes into measurable optical signals through three primary scattering mechanisms and engineered structures:

- Rayleigh scattering from random refractive index fluctuations forms the basis of optical time-domain reflectometry (OTDR) and DAS [22,23,24].
- Brillouin scattering from light–acoustic interactions produce frequency shifts linearly dependent on strain and temperature.
- Raman scattering from molecular vibrations enables distributed temperature sensing through the anti-Stokes/Stokes intensity ratio.

- Fiber Bragg gratings (FBGs) (Figure 4) impose periodic refractive index modulation, creating wavelength-selective reflectors where the Bragg wavelength $\lambda_{B}=2n_{eff}\Lambda$ shifts with strain and temperature.

Typical FBGs are fabricated in germanium-doped silica fibers (GeO_2_-SiO_2_) with effective refractive indices $n_{eff}$ ranging from 1.447 to 1.452 at 1550 nm. Pure silica cores (SiO_2_) yield $n_{eff}$ ≈ 1.444, while higher doping concentrations (e.g., 20 mol% GeO_2_) can increase $n_{eff}$ to approximately 1.46. The photosensitivity required for grating inscription is enhanced through hydrogen loading or boron co-doping, which increases the refractive index modulation depth without significantly altering $n_{eff}$.

**Figure 5:**
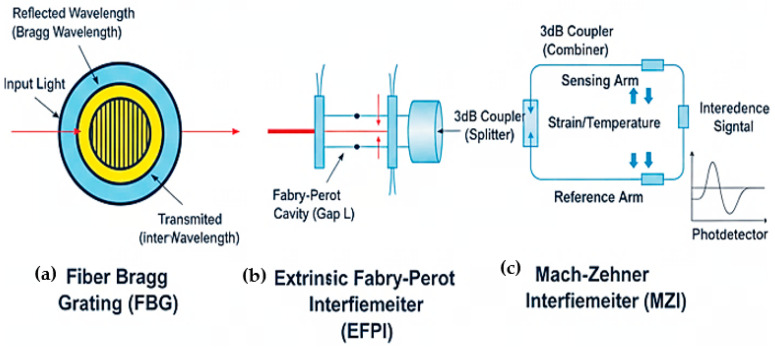
Point sensor configurations: (**a**) Single FBG sensor. (**b**) Extrinsic Fabry–Perot sensor. (**c**) Intensity-based point sensor with reflective target [26].

Sensor Classification: OFS architectures span three spatial regimes, Figure 5: point sensors (localized measurements at discrete locations), quasi-distributed sensors (FBG arrays addressed via wavelength- or time-division multiplexing) [23], and fully distributed sensors (continuous sensing along the entire fiber length using scattering mechanisms). Transduction approaches include intensity, wavelength, phase, and polarization modulation, each offering distinct sensitivity and multiplexing trade-offs.

### 3.2. Performance Metrics and Characterization Framework

#### Fundamental Performance Parameters

Comprehensive characterization of OFS requires evaluation across multiple performance metrics that collectively define system capabilities and limitations [27,28]. Table 1 summarizes key performance parameters that describe the operational characteristics of OFS systems.

Sensitivity refers to the magnitude of output change per unit variation in the measured quantity. It varies with sensor technology and depends on factors such as the transduction mechanism and the interrogation method used. Resolution denotes the smallest detectable change in the measurand, typically on the order of 1 με for FBG sensors and 0.1 °C for DTS, and is governed primarily by the signal-to-noise ratio (SNR) and source stability [29]. Spatial resolution indicates the minimum distinguishable distance between sensing points, ranging from about 1 m in DTS to 1 cm in DAS, and is determined by pulse width and the underlying scattering mechanism.

Dynamic range represents the ratio between the maximum and minimum detectable signal levels, typically between 40 and 60 dB, and is influenced by detector linearity and the system noise floor. Accuracy measures how closely the sensor output matches the true value (e.g., ±1 °C for DTS and ±2 με for FBG) and depends on calibration precision and cross-sensitivity effects. Finally, cross-sensitivity reflects unwanted responses to non-target parameters, such as temperature–strain coupling, which are influenced by material properties and sensor design.

Together, as presented in Table 1, these parameters provide a comprehensive framework for evaluating and optimizing the performance of OFS technologies.

**Table 1:** Key performance metrics for optical fiber sensors.

Parameter	Definition	Typical Range	Primary Dependencies
Sensitivity	Output change per unit measurand	Technology dependent [30]	Transduction mechanism, interrogation method
Resolution	Minimum detectable change	1 με (FBG), 0.1 °C (DTS) [31]	SNR, source stability
Spatial Resolution	Minimum distinguishable distance	1 m (DTS) to 1 cm (DAS) [32]	Pulse width, scattering mechanism
Dynamic Range	Maximum to minimum detectable ratio	40–60 dB [33]	Detector linearity, system noise floor
Accuracy	Deviation from true value	±1 °C (DTS), ±2 με (FBG) [34]	Calibration precision, cross-sensitivity
Cross-Sensitivity	Unwanted parameter response	Temperature–strain coupling [35]	Material properties, sensor design

### 3.3. Fundamental Limitations and Technical Challenges

The performance of OFS systems is fundamentally limited by Rayleigh noise, nonlinear effects such as stimulated Brillouin scattering (SBS) and four-wave mixing (FWM), and intrinsic material attenuation. Additional challenges arise from cross-sensitivity, multiplexing constraints, and increasing computational demands associated with large-scale, high-resolution sensing. Future systems are expected to integrate SDM and AI-driven analytics to overcome these limitations, enabling enhanced scalability, robustness, and intelligence in optical fiber sensing networks.

**Figure 6:**
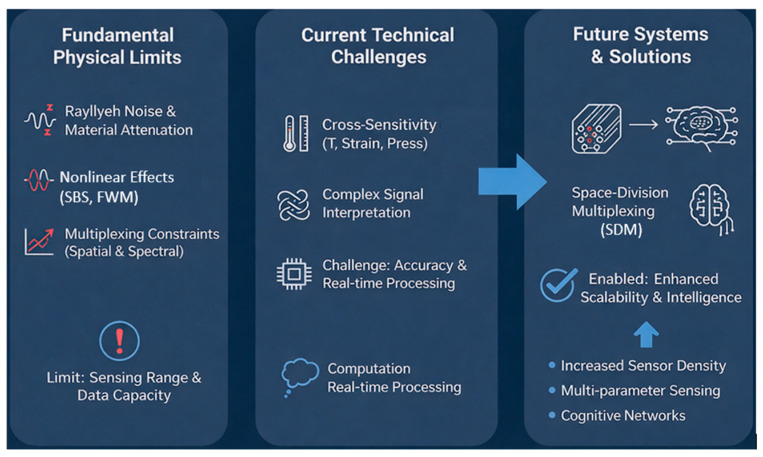
Fundamental performance limitations and technical challenges in optical fiber sensing [37].

Figure 6 presents an overview of the evolution and future direction of optical fiber sensing technologies, structured into three key domains: fundamental physical limits, current technical challenges, and future systems and solutions. The first section highlights intrinsic constraints such as Rayleigh noise and material attenuation, nonlinear optical effects (including stimulated Brillouin scattering and four-wave mixing), and multiplexing limitations in spatial and spectral domains, all of which restrict sensing range and data capacity [36].

The second section identifies ongoing challenges, including temperature, strain, and pressure cross-sensitivity; complex signal interpretation; and difficulties in maintaining high accuracy and real-time data processing under computational constraints [38]. The final section outlines emerging strategies aimed at overcoming these barriers, emphasizing SDM and intelligent, multi-parameter cognitive sensor networks that enhance scalability, sensing density, and overall system intelligence [39]. Collectively, these highlight the technological transition from physically constrained sensing architectures toward advanced, data-driven, and scalable intelligent sensing networks.

## 4. Optical Fiber Sensors: Foundations and Scalability Challenges

### 4.1. The Rise of OFS: Enabling Attributes and Application Domains

OFS emerged from the telecommunications revolution of the late 20th century to become a cornerstone technology for measuring physical, chemical, and biological parameters [40,41]. Their widespread adoption stems from five inherent advantages that distinguish them from conventional electronic sensors:

- Immunity to EMI: As dielectric waveguides, optical fibers are unaffected by lightning, high-voltage equipment, or radio frequencies, enabling reliable operation in electrically noisy environments such as power plants and industrial facilities [36].
- Intrinsic Safety: Operation at low optical power with no spark generation makes OFS ideal for hazardous and explosive atmospheres prevalent in oil, gas, and mining industries [36].
- Small Size and Lightweight: The slender, flexible form factor enable minimally invasive embedding into composite materials, concrete structures, and wearable devices, creating “smart materials” or “nervous systems” for infrastructure monitoring [42].
- Multiplexing Capability: Multiple sensing points or continuous sensing regions can be implemented along a single fiber, drastically reducing per-sensor cost and complexity compared to electronic arrays [43].
- Remote and Distributed Sensing: Measurements over tens of kilometers enable monitoring of extensive, remote, or inaccessible infrastructure including pipelines, borders, and power cables [44].

These attributes have established OFS as the technology of choice for structural health monitoring of bridges, tunnels, and dams [45,46]; downhole monitoring in oil and gas wells [45]; perimeter intrusion detection [47]; and biomedical applications for in vivo sensing and minimally invasive surgery [48].

### 4.2. The Multiplexing Imperative and the Scalability Challenge

Modern sensing applications demand dense spatial data—knowing that a bridge is under stress is insufficient; pinpointing the exact location and magnitude is critical. This imperative has driven the development of multiplexing techniques that maximize data yield per installed fiber [49].

#### Classical Multiplexing Domains

- Time-Division Multiplexing (TDM): Sensors are addressed by optical pulse time-of-flight, forming the foundation for DAS, Brillouin-based sensing (DBS), and distributed temperature sensing (DTS) [50]. However, spatial resolution Δz is inversely proportional to pulse width τ (Δz ∝ cτ/2n), while range L is limited by attenuation (L ∝ 1/α), creating a fundamental trade-off [51,52].
- Wavelength-Division Multiplexing (WDM): FBGs operate at distinct wavelength bands within the available optical spectrum [53]. Capacity is constrained by source bandwidth and the ITU grid, typically limiting networks to 40–80 sensors per fiber [54].
- Frequency-Division Multiplexing (FDM): Less common, this approach modulates sensors at distinct radio frequencies but faces similar scalability constraints [55].

The Scalability Bottleneck:

Despite commercial success, these techniques are approaching fundamental physical limits [56]. Escalating demands for higher sensor density, finer spatial resolution (<10 cm), and simultaneous multi-parameter measurement in complex environments have exposed a critical bottleneck [57]. Deploying multiple parallel SMF, a brute-force solution, increases cost, weight, and cabling complexity, undermining a key advantage of OFS.

**Figure 7:**
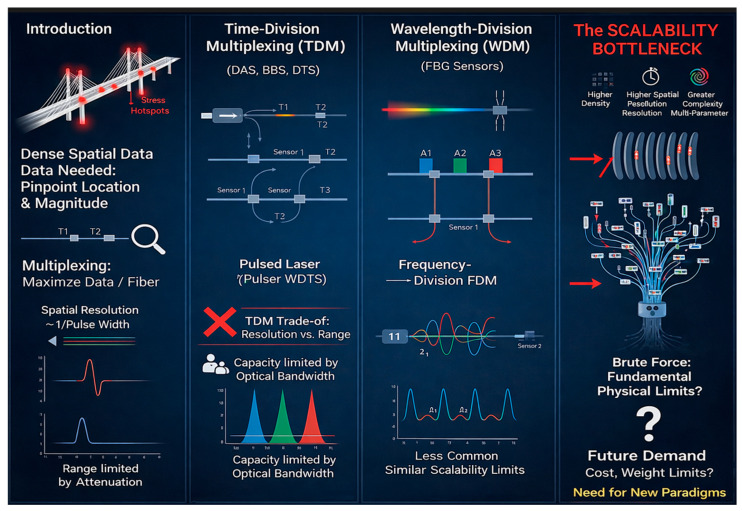
Overview of multiplexing techniques in OFS, comparing TDM, WDM, and FDM methods, their resolution–range trade-offs, and scalability limits, while highlighting the need for new paradigms to overcome bandwidth and physical constraints [58].

Figure 7 synthesizes these multiplexing strategies, illustrating the resolution–range trade-off in TDM, the finite bandwidth constraint in WDM, and the resulting imperative for new models. Overcoming these limits requires exploiting the spatial dimension of the fiber itself, the central premise of SDM detailed in the subsequent sections.

### 4.3. An SDM: A Paradigm Shift in Capacity

To overcome this bottleneck, the field has turned to a model that has already revolutionized optical communications: an SDM [59,60,61]. The core premise of SDM is simple yet powerful: to exploit the spatial dimension of the optical fiber itself as a new resource for multiplexing. Instead of using a single core within the fiber’s cladding, SDM employs specialized fiber geometries to create multiple, parallel optical pathways [62].

**Figure 8:**
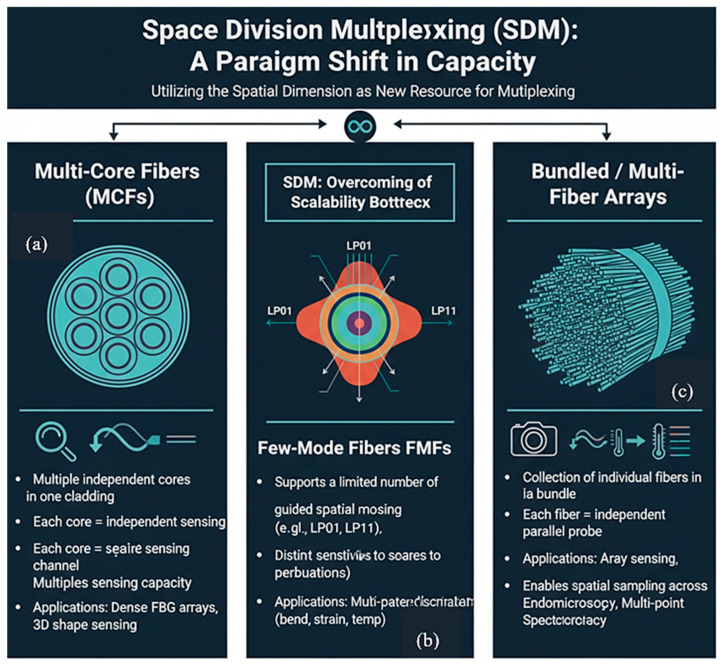
Microscopic images of SDM fibers: (**a**) SEM image of a 7-core MCF (125 μm cladding, scale bar: 20 μm); (**b**) optical microscope image of a 4-mode FMF (20 μm core) with mode field distributions (LP_01_, LP_11_, LP_21_, LP_02_) and refractive index profile (inset) [65]; (**c**) packaged multi-fiber array for 3D shape reconstruction [70].

The three primary SDM approaches relevant to sensing are:

- Multi-Core Fibers (MCFs): These fibers incorporate multiple independent cores within a single cladding [14]. Each core can function as an independent sensing channel, effectively multiplying the sensing capacity of a single fiber by the number of cores. This is particularly powerful for creating dense arrays of FBGs or for distributed sensing with inherent spatial diversity (Figure 8a), enabling applications like full 3D shape sensing [63].
- Few-Mode Fibers (FMFs): These fibers are designed to support the propagation of a limited number of guided spatial modes (e.g., LP01, LP11, etc.) [62,64,65]. In this approach, known as MDM, each spatial mode can be exploited as a separate sensing channel (Figure 8b). Crucially, different modes exhibit distinct sensitivities to external perturbations like bend, strain, and temperature, opening the door to sophisticated multi-parameter discrimination from a single fiber [66].
- Bundled/Multi-Fiber Arrays: This approach involves bundling a collection of individual optical fibers into a coherent array. Each fiber acts as an independent parallel fiber probe, enabling spatial sampling across a surface. This configuration is fundamentally used for array sensing (Figure 8c), where data is collected simultaneously from multiple discrete points [67]. A prominent application is in medical imaging, particularly in endomicroscopy, where a thin, flexible fiber bundle can be inserted into the body to transmit an image from the internal tissue to an external camera [68,69]. It is also used in spectroscopy for multi-point analysis.

SDM represents a quantum leap in scaling potential, promising orders-of-magnitude increases in data density and parallelism [71]. However, this leap comes with its own set of formidable challenges, including inter-core and inter-mode crosstalk [72], increased complexity in signal interrogation and demultiplexing [73], and specialized fabrication requirements [74]. Table 2 categorizes the primary SDM fiber types, outlining their core principles, key sensing opportunities, and inherent challenges, illustrating a progression from simple data parallelization to advanced multi-parameter extraction.

In summary, Table 2 illustrates a clear evolution in sensing capability:

- FMFs represent a paradigm shift towards sophisticated multi-parameter extraction by leveraging the unique physical properties of different light paths (modes) within the same core [75].
- MCFs move beyond simple parallelization by offering spatially co-located sensing points, enabling redundancy and basic multi-parameter analysis [76].
- Bundled fibers offer the simplest form of parallelization with high channel isolation but sacrifice the spatial co-location and advanced physical insights offered by the more integrated MCF and FMF approaches [69].

**Table 2:** SDM fiber types, outlining their core principles, key sensing opportunities, and inherent challenges.

SDM Fiber Type	Core Principle & Structure	Key Sensing Opportunities	Primary Challenges
MCF	Multiple single-mode cores in a common cladding.	Parallel multi-point sensing with core-specific FBG arrays [76]. Multi-parameter/redundant sensing using functionalized or heterogeneous cores [77].	Inter-core crosstalk affecting accuracy [15,77]. Complex and costly fabrication/splicing [75]. Larger diameter reduces flexibility.
FMF	Single large core supporting few spatial modes (e.g., LP_01_, LP_11_).	Multi-parameter sensing (strain, temperature, bend) via modal diversity [75]. ML-assisted modal separation [78,79].	Mode coupling and signal distortion. Complex MIMO-DSP interrogation [80]. Requires mode-selective components.
Bundled/Multi-Fiber Array	Multiple discrete SMFs bundled in one sheath [81].	High isolation and scalable sensing using standard fibers [82,83]. Low-cost and flexible deployment.	Bulky structure with reduced flexibility. Inter-fiber calibration and non-co-located sensing issues.

### 4.4. The Data Deluge and the Need for Intelligence

The implementation of SDM inevitably generates an explosion in data volume and complexity. For example, a single MCF with seven cores performing DAS produces approximately a sevenfold increase in data compared to a standard single-core fiber [84]. Furthermore, disentangling the combined signals from multiple cores or modes and accurately inverting this data into precise physical measurands is a highly complex, often nonlinear problem that pushes traditional signal processing algorithms to their limits [82]. This “big data” challenge coincides with the rise of AI and, more specifically, ML and DL, which have demonstrated remarkable capabilities in pattern recognition, noise suppression, and modeling complex nonlinear systems [85]. AI is no longer merely a useful tool but is becoming an indispensable component of advanced OFS [86]. Its potential applications are manifold: from denoising weak scattering signals [87,88] and classifying acoustic events in DAS data [89] to solving inverse problems involved in shape sensing and multi-parameter discrimination [90].

**Figure 9:**
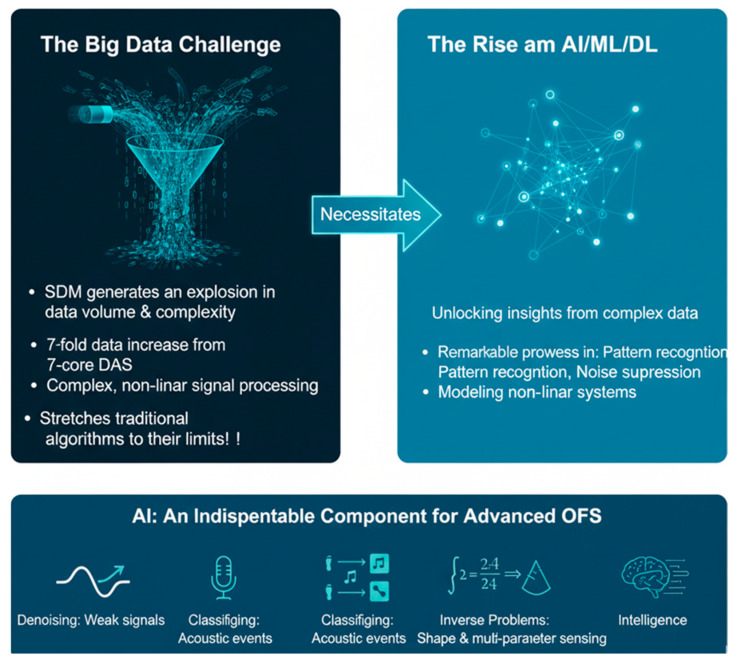
Data deluge and the need for intelligent SDM sensor embracing advantage of ML and DL [92].

The illustration in Figure 9 demonstrates the symbiotic relationship between SDM sensors and AI in advancing OFS technologies. The left panel highlights the big data challenge, where SDM dramatically increases data volume and complexity, such as a sevenfold rise in data from seven-core DAS, resulting in highly nonlinear, multidimensional signal processing demands that push conventional algorithms to their limits [91].

The middle section emphasizes that this surge in data necessitates the adoption of AI, ML, and DL techniques to extract meaningful insights from complex datasets [93]. The right and bottom sections collectively demonstrate AI’s transformative capabilities in pattern recognition, noise suppression, and nonlinear modeling, enabling advanced tasks such as denoising weak signals, classifying acoustic events, solving inverse problems, and enhancing multi-parameter sensing and system intelligence [21,94]. Overall, the diagram underscores AI as an indispensable enabler for overcoming SDM-driven data challenges and achieving intelligent, high-performance OFS systems.

### 4.5. The Confluence: A New Frontier for Intelligent Sensing

It is at the intersection of these two transformative trends (SDM and AI) that a new frontier in optical fiber sensing is emerging. SDM provides a rich, multidimensional spatial canvas, while AI provides the computational intelligence to interpret it. This synergistic relationship is bidirectional:

- AI empowers SDM-OFS by mitigating its inherent challenges (e.g., using NN for digital crosstalk suppression [95] and unlocking its full potential (e.g., using DL for super-resolution sensing [96]).
- SDM-OFS empowers AI by providing the high-dimensional, feature-rich data required to train robust and accurate models [77].

**Figure 10:**
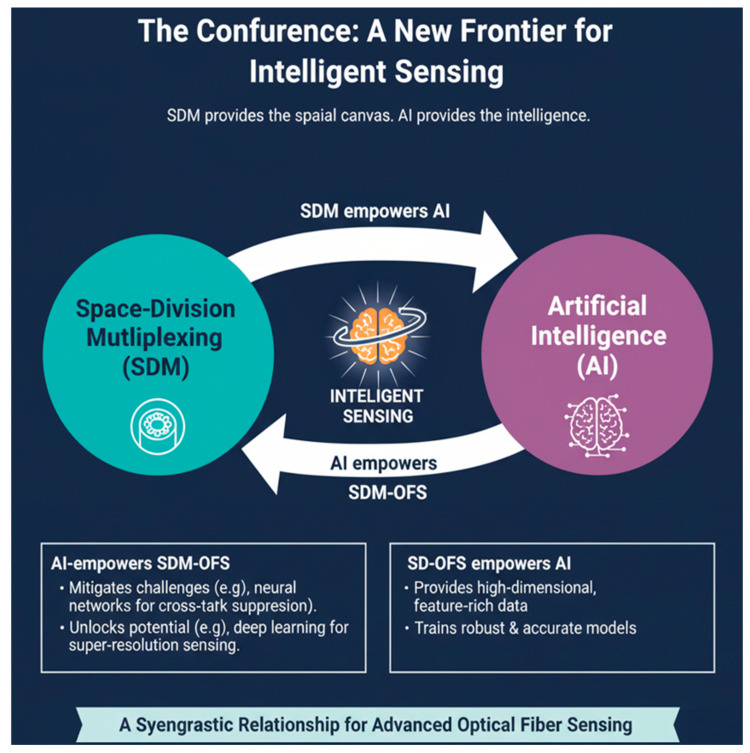
AI empowers SDM-OFS, mitigating challenges and unlocking potential [86].

Figure 10 illustrates the synergistic convergence between SDM and AI as a transformative model for intelligent OFS. It conceptualizes a bidirectional relationship in which SDM empowers AI by providing high-dimensional, feature-rich data from multi-core or multi-mode fibers, thereby enabling the development of robust and accurate ML models [97]. Conversely, AI empowers SDM-OFS by addressing inherent challenges such as crosstalk suppression, noise reduction, and nonlinear signal processing through advanced techniques such as DL and neural network modeling [21,94]. This mutual reinforcement, depicted as a feedback loop, shows how SDM offers the spatial canvas while AI provides the intelligence needed to extract meaningful insights, leading to enhanced sensitivity, resolution, and multi-parameter sensing capabilities [98]. Overall, the illustration highlights a synergistic frontier for intelligent sensing, establishing AI as an indispensable enabler for the next generation of SDM-based optical fiber systems.

**Figure 11:**
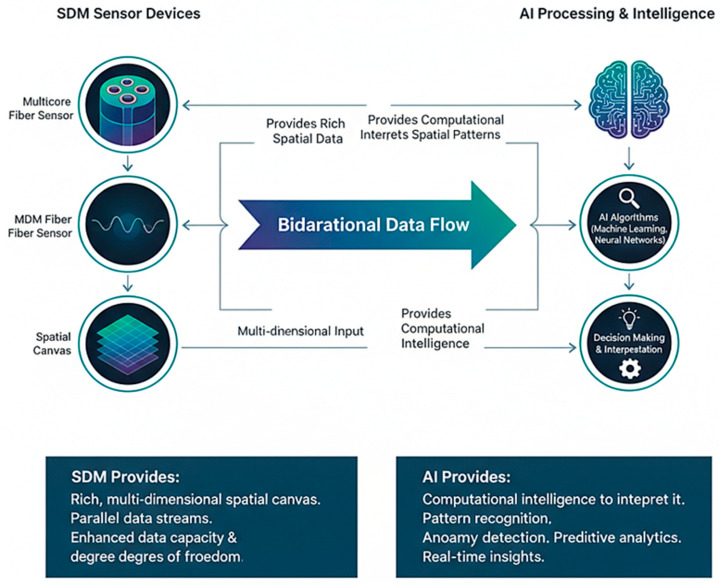
Synergetic bidirectional relationship between SDM and AI: A unified intelligence framework [100].

Figure 11 illustrates the bidirectional data flow between SDM sensor devices and AI-based processing and intelligence, emphasizing their complementary roles in advancing modern OFS systems. On the left, multi-core, few-/multi-mode, and spatially multiplexed fibers generate rich, multidimensional spatial data, providing parallel data streams with enhanced sensing capacity and degrees of freedom [99].

This high-dimensional input is transmitted to the AI domain, where ML and neural network algorithms interpret complex spatial patterns, perform pattern recognition and anomaly detection, and deliver real-time decision-making and predictive analytics [101,102]. The process is reciprocal, as AI not only interprets data but also feeds back computational intelligence that optimizes sensing configurations, noise suppression, and event classification [103]. Overall, the diagram highlights the synergistic integration of SDM and AI, where SDM provides the spatial canvas, and AI contributes the computational intelligence, together forming a closed-loop architecture for intelligent, adaptive, and high-capacity optical sensing systems.

This provides strong motivation to explore this powerful convergence. In the subsequent sections, the state of the art in SDM-based sensing will be dissected, the application of AI techniques to enhance these systems will be critically analyzed, and the future trajectory of this rapidly evolving field will be projected, pointing toward a new generation of intelligent, high-dimensional sensing networks.

## 5. Artificial Intelligence in OFS

The advent of high-resolution, distributed, and multi-parameter OFS systems has generated a paradigm shift from data-scarce to data-rich sensing. Systems such as DAS and high-density Bragg grating arrays produce vast, complex datasets that are often nonstationary and contain subtle, nonlinear signatures. Traditional signal processing and analytical models, while effective for simpler systems, struggle to handle this scale and complexity [94]. This has positioned AI and ML not merely as useful tools but as foundational technologies for unlocking the full potential of advanced OFS. This section examines the role of AI in managing this complexity, outlines the key ML paradigms employed, and reviews their transformative applications in conventional OFS systems, thereby setting the stage for their critical role in the forthcoming discussion on SDM sensing.

**Figure 12:**
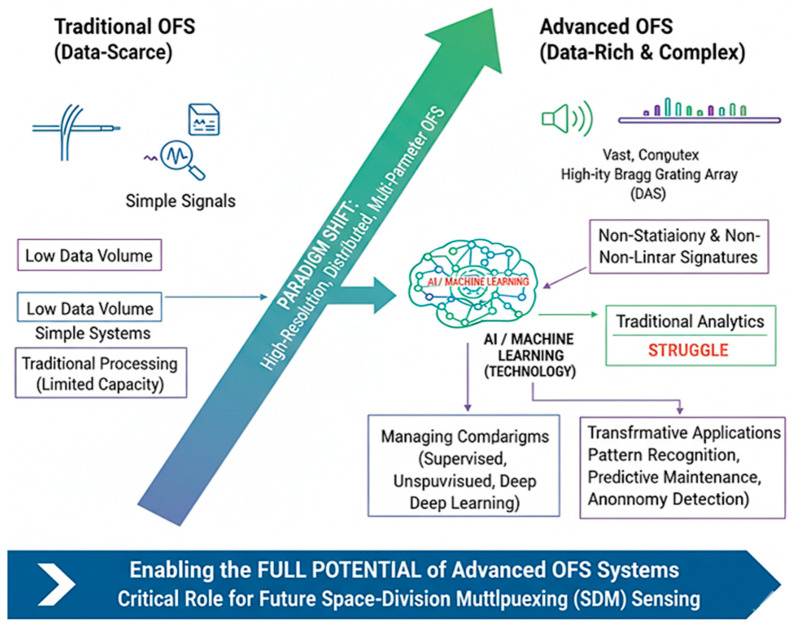
Optical fiber sensing: From data-scarce to data-rich regimes [99].

As illustrated in Figure 12, the paradigm shift from traditional data-scarce OFS systems to advanced, data-rich, and complex OFS architectures is driven by the integration of AI and ML. Traditional OFS systems, characterized by low data volume, simple signals, and limited analytical capacity, are increasingly inadequate for modern sensing needs [101]. In contrast, advanced OFS systems, such as high-fidelity Bragg grating arrays and DAS, generate massive, nonstationary, and nonlinear datasets that exceed the capabilities of conventional processing techniques [75].

These underscore AI/ML as a transformative enabler, capable of managing complex data models through supervised, unsupervised, and deep learning approaches while enabling advanced applications including pattern recognition, predictive maintenance, and anomaly detection [104,105]. This shift empowers the realization of high-resolution, distributed, and multi-parameter sensing, marking a critical step toward unlocking the full potential of next-generation OFS systems through SDM and AI-assisted data analytics.

### 5.1. The Indispensability Theory: Why AI Is Fundamentally Necessary for SDM Sensing

The convergence of SDM and AI represents more than an incremental improvement; it addresses a fundamental mathematical and physical reality: SDM-generated data exists in a dimensionality that exceeds the capacity of classical signal processing to fully exploit. This indispensability rests on three foundational arguments:

#### The Dimensionality–Information Paradox

SDM sensing generates data across multiple spatial channels (cores or modes) simultaneously [97]. For an MCF with N cores, each producing an M measurement in points per second, the resulting data tensor exists in ℝ^(N × M × T). Classical signal processing operates effectively in low-dimensional spaces but suffers from the “curse of dimensionality” when attempting to extract information from such high-dimensional manifolds. AI, particularly DL, is uniquely equipped to model probability distributions in high-dimensional spaces through hierarchical feature learning. Without AI, the information contained in cross-channel correlations, precisely the rich data that makes SDM valuable, remains largely inaccessible to conventional algorithms.

### 5.2. Mathematical Framework

#### 5.2.1. Mathematical Formulation of the SDM Inverse Problem

The forward problem in SDM sensing establishes the relationship between the physical quantities to be measured and the optical signals captured by the interrogation system. This relationship can be expressed as [106]

(1) y=F(x)+n

where the symbols are defined as follows:

y represents the measured or observed optical signal obtained from the interrogation system. F(⋅)—Forward operator or system response function that maps the physical quantities to the measured optical signal. It represents the physical sensing process, including light propagation and interaction with the sensing medium. x denotes the vector of physical parameters or measurands to be estimated (e.g., temperature, strain, pressure, or refractive index variations along the fiber). n is the measurement noise introduced by the sensing system, such as detector noise, environmental disturbances, and system imperfections. This relation represents the forward sensing model, where the measured signal y is produced by the transformation of the physical parameters x through the system operator F(⋅), with additional noise n. This model forms the basis for solving the inverse problem, where the goal is to estimate x from the observed measurements y.

#### 5.2.2. Coupled-Mode Equations for Multi-Core Fibers

For coupled-core multi-core fibers (CC-MCFs) with *K* cores, the propagation of optical fields is governed by the coupled-mode equations [107]:

(2) $$\frac{dA}{dz}=-jCA+jB(z)A$$

The terms in the equation are defined as follows:

A—Complex field amplitude vector representing the optical signals propagating in the K cores of the multi-core fiber. It is typically expressed as

$$A=[A_{1},A_{2},\ldots ,A_{K}{]}^{T}$$

where $A_{k}$ is the optical field in the k-th core. $\frac{dA}{dz}$ represents the derivative of the optical field vector with respect to the propagation distance z, describing how the optical fields evolve along the fiber. Also, z denotes the longitudinal propagation coordinate along the fiber.

j—Imaginary unit $\left(j=\sqrt{-1}\right)$, commonly used in optical and electrical engineering.

C—Coupling matrix that characterizes the linear coupling between the different cores of the multi-core fiber. The off-diagonal elements represent inter-core coupling coefficients, while the diagonal elements may represent propagation constants.

B(z)—A position-dependent matrix representing perturbations along the fiber, such as birefringence, phase mismatch, or random propagation variations, and K is the number of cores in the CC-MCF system.

Equation (2) describes the evolution of the optical field vector along MCF, where the first term—jCA—represents deterministic coupling between the cores, and the second term—jB(z)A—accounts for position-dependent perturbations or phase variations in the fiber. 

#### 5.2.3. The Inverse Problem

Recovering the physical parameters from the measured optical data requires solving the inverse problem [106]:

(3) $$\hat{x}=F^{-1}(y)$$

$\hat{x}$ denotes the estimated or reconstructed signal obtained after applying the inverse transformation or recovery process. $F^{-1}(\cdot )$ is the inverse operator or inverse transformation of the forward model F(⋅). It represents the process used to recover the original signal from the observed data. y is the observed or measured signal, typically affected by channel distortion, noise, or system impairments. Thus, the equation expresses the signal reconstruction process, where the estimated signal, $\hat{x},$ is obtained by applying the inverse mapping $F^{-1}$ to the observed signal y. In practical systems, this inverse operation may be implemented using digital signal processing or a learning-based model to compensate for channel distortions.

#### 5.2.4. AI-Based Solution Approach

ML methods address the ill-posed inverse problem by learning an approximation of the inverse mapping [108]:

(4) $$G_{\theta }(y)\approx F^{-1}(y)$$

$G_{\theta }$ is the parameterized model (e.g., an NN or learning-based equalizer) used to approximate the inverse mapping of the system. θ denotes trainable parameters of the model $G_{\theta }$, such as weights and biases. y is the observed or received signal that has been distorted by the channel or system impairments. $F^{-1}(\cdot )$ represents the true inverse operator of the system or channel transformation F(⋅), which ideally recovers the original signal from the distorted observation.

In essence, Equation (4) states that the learned model $G_{\theta }(y)$ is trained to approximate the inverse system function $F^{-1}(y)$, enabling recovery or reconstruction of the original signal from the distorted observation y. This approach is common in data-driven equalization or inverse problem solving, where the exact analytical inverse may be difficult or impossible to compute.

Equation (5) represents the regularized empirical risk minimization problem [109]. The optimal parameters $\theta^{*}$ are obtained by minimizing the total loss consisting of the prediction error between the model output $G_{\theta }(y_{i})$ and the true signal $x_{i}$, along with a regularization term that constrains the model complexity.

(5) $$\theta^{*}=arg\;{min}_{\theta }\sum_{i=1}^{L}∥G_{\theta }(y_{i})-x_{i}{∥}_{2}^{2}+\lambda R(\theta )$$

where $\theta^{*}$ is the optimal set of model parameters obtained after training.

$\arg{min}_{\theta }$ are the parameter values θ that minimize the objective (loss) function.

θ—Trainable parameters of the model (e.g., neural network weights and biases). L represents the total number of training data samples used for model optimization.

$y_{i}$ denotes the i-th observed or distorted input signal (e.g., received signal).

$x_{i}$ is the corresponding i-th target or reference signal (e.g., transmitted or clean signal). $G_{\theta }(\cdot )$ represents the parametric model or NN that maps the input $y_{i}$ to an estimated output, parameterized by θ. $∥\cdot {∥}_{2}^{2}$ is t-squared $L_{2}$-norm representing the squared error between the predicted output and the target signal.

λ—Regularization coefficient controlling the influence of the regularization term. R(θ) denotes the regularization function applied to the parameters (e.g., $L_{2}$ weight penalty) to prevent overfitting and improve generalization.

#### 5.2.5. Physics-Informed Neural Networks (PINNs)

For PINNs, the loss function incorporates both data fidelity and physical consistency [110]:

(6) $$L(\theta )=L_{\mathrm{data}}(\theta )+\lambda L_{\mathrm{physics}}(\theta )$$

where L(θ) is the total loss function used during the training of the model. It combines the data-driven error and the physics-based constraint. θ represents the trainable parameters of the model, such as the weights and biases of the neural network. $L_{\mathrm{data}}(\theta )$ denotes the data loss term that measures the discrepancy between the model predictions and the observed or labeled data (e.g., mean squared error between predicted and true signals). $L_{\mathrm{physics}}(\theta )$ is the physics-based loss term that enforces consistency between the model output and the governing physical equation of the system. λ is the regularization or weighting coefficient that balances the contribution of the physics-based loss relative to the data loss.

The total loss function combines data-driven learning and physics-informed constraints. The parameter λ  controls how strongly the physical model influences the training compared to the measured data.

The physics-based loss enforces consistency with the coupled-mode theory [110]:

(7) $$L_{\mathrm{physics}}(\theta )=\sum_{j=1}^{J}{∥\frac{dA_{\theta }(z_{j})}{dz}+jCA_{\theta }(z_{j})-jB(z_{j})A_{\theta }(z_{j})∥}_{2}^{2}$$

The additional symbols in Equation (7) are defined as:

$L_{\mathrm{physics}}(\theta )$—Physics-based loss function used to enforce the governing physical equation during model training. J is the total number of collocation or sampling points used to evaluate the physical constraint. $z_{j}$ is the j-th spatial coordinate (e.g., propagation distance along the optical link). $A_{\theta }(z_{j})$ denotes model-predicted complex signal envelope (or optical field amplitude) at position $z_{j}$, parameterized by θ. $\frac{dA_{\theta }(z_{j})}{dz}$ is the spatial derivative of the predicted signal envelope with respect to the propagation coordinate z.

C represents the linear coefficient representing attenuation or gain in the propagation medium. $B(z_{j})$ is the spatially varying coefficient describing phase distortion, turbulence, or other propagation-dependent effects.

The PINN approach described by Equations (6) and (7) provides a systematic framework for incorporating physical knowledge into the learning process. By minimizing the residual of the coupled-mode equations alongside the data fidelity term, PINNs ensure that the learned inverse mapping respects the underlying physics, improving generalization and reducing data requirements. Table 3 depicts the mathematical formulation summary used in the proposed PINN framework for SDM sensing in CC-MCFs.

#### 5.2.6. The Inverse Problem Complexity

SDM sensing fundamentally requires solving ill-posed inverse problems: inferring physical perturbations (strain, temperature, bend) from measured optical responses. In single-mode fibers, this inverse mapping is challenging but often tractable. In SDM systems, the mapping becomes:

- For MCFs: Each core responds to perturbations, but with core-specific sensitivities and inter-core coupling.
- For FMFs: Multiple spatial modes exhibit distinct perturbation responses, and mode coupling creates complex superposition states.
- For coupled-core fibers: Supermode field distributions respond collectively to perturbations, creating a nonlinear mapping from N physical parameters to K supermode states.

The forward model F: 𝒫 → 𝒮 (physical parameters to signal space) is known approximately, but its inverse F^−1^: 𝒮 → 𝒫 is non-unique, nonlinear, and often analytically intractable. AI provides a data-driven approach to learning approximations of F^−1^ without requiring explicit analytical inversion, making it not merely helpful but essential for practical SDM deployment.

#### 5.2.7. The Real-Time Processing Constraint

SDM systems generate data at rates exceeding conventional processing capabilities. A 32-core DAS system sampling at 10 kHz with 8-bit resolution produces approximately 9.2 GB/hour—exceeding real-time processing capacity of traditional DSP pipelines. AI enables:

- Dimensionality reduction at the edge before transmission;
- Event-triggered processing rather than continuous analysis;
- Learned compression that preserves salient features.

Without AI-enabled edge processing, the data volume alone would render many SDM applications impractical for real-world deployment.

#### 5.2.8. Synthesis: The Symbiotic Necessity

The relationship is therefore not optional enhancement but symbiotic necessity: SDM provides the spatial dimensionality that classical methods cannot fully exploit, while AI provides the computational architecture designed explicitly for high-dimensional inference. This mutual dependence defines the indispensability thesis that frames the remainder of this review.

### 5.3. Problem-Centric Mapping: AI Models for SDM Physical Challenges

Rather than cataloging algorithms, this section maps unique SDM physical challenges to specific AI paradigms with mechanistic explanations (Table 4).

#### Detailed Analysis: From Physics to Algorithm

Inter-core crosstalk in MCFs arises from evanescent field coupling between adjacent cores, governed by coupled-mode equations [110]:

(8) $$\frac{dA_{i}}{dz}=-j\sum_{k\neq i}\kappa_{ik}A_{k}e^{j(\beta_{i}-\beta_{k})z}$$

where *κ_ik_* is the coupling coefficient. Traditional compensation requires precise knowledge of *κ_ik_* and its environmental dependence is practically impossible. DNNs learn the nonlinear mapping A_measured = f (A_true, environmental parameters) through supervised training on known perturbation scenarios, effectively learning the inverse coupling function without explicit physical modeling.

Inter-mode coupling in FMFs creates complex superposition states where the measured field E_measured = Σ c_m_(z)ψ_m_ e^(jβ_m_z) with random coupling coefficients c_m_(z). Complex-valued NNs preserve both amplitude and phase information, learning to recover individual mode coefficients from measured interference patterns. This preserves the phase information essential for coherent sensing while handling the stochastic nature of mode coupling.

Multi-parameter cross-sensitivity is the fundamental challenge that temperature and strain produce similar spectral shifts and becomes addressable through attention mechanisms that learn to weight contributions from different modes/cores based on their distinct sensitivity profiles. This transforms a previously ill-posed inverse problem into a well-posed learned mapping [109].

### 5.4. Quantitative Benchmarking: AI-Enabled Performance Improvements

To enable meaningful evaluation of AI’s impact on SDM sensing, Table 5 synthesizes results from representative studies, reporting standardized performance metrics and improvement factors over conventional methods.

Key Observations

- SNR improvements: 9–10 dB through crosstalk compensation and denoising.
- Spatial resolution: 6× enhancement beyond physical limits.
- Multi-parameter discrimination: 3–5× improvement via multi-task and attention learning.
- Classification accuracy: 10–15% gains for event detection.
- Latency reduction: 10–20× through edge-deployed lightweight models.

These quantitative results demonstrate order-of-magnitude improvements across critical dimensions, supporting the indispensability thesis.

### 5.5. Key Machine Learning Paradigms for OFS

The ML landscape applied to OFS is diverse, with different models suited to specific tasks, as shown in Table 6. The primary categories are:

o Supervised Learning: This is the most prevalent paradigm, used when a labeled dataset is available. The model learns a mapping from input data to known output labels.
o Convolutional Neural Networks (CNNs) are predominantly used for image-like data. In OFS, DAS data is often represented as spatiotemporal maps (distance–time), where CNNs excel at classifying events such as footsteps, vehicles, or drilling [121].
▪ Deep Neural Networks (DNNs) for regression are used to learn continuous functions. They are widely applied to solve inverse problems, such as estimating temperature or strain values from a complex interference spectrum or FBG reflection profile [86].

Supervised Learning dominates structured sensing tasks. CNNs excel with spatiotemporal DAS data (distance–time maps) for classifying footsteps, vehicles, or drilling events [125]. DNN regression solves inverse problems—estimating temperature or strain from complex interference spectra or FBG reflection profiles [126].

Unsupervised Learning addresses scenarios with scarce labeled data. Clustering algorithms (K-means, DBSCAN) enable anomaly detection by learning “normal” system states and flagging deviations—critical for infrastructure health monitoring [127].

Deep Learning extends capabilities through specialized architectures:

- Autoencoders perform unsupervised dimensionality reduction and denoising, reconstructing clean signals from noisy inputs to enhance SNR [128].
- RNNs and LSTMs analyze time-series data for temperature trend prediction, vibration pattern classification, and transient acoustic event detection [129].

The integration of these paradigms transforms OFS from data collection tools into intelligent systems for automated insight generation. Figure 12 illustrates this paradigm shift: overcoming high-dimensional data, nonlinear physics, low SNR, and real-time demands through DNNs, automated pattern recognition, and edge AI inference [130].

Summary: AI provides not marginal but transformative improvements for SDM sensing. Problem-centric mapping (Table 4) links physical challenges to appropriate AI paradigms, while quantitative benchmarking (Table 5) demonstrates order-of-magnitude gains across SNR, resolution, accuracy, and latency—establishing AI as indispensable for practical SDM deployment.

## 6. The Synergy: AI-Assisted SDM Optical Fiber Sensors

AI-enabled SDM optical fiber sensing constitutes a transformative advance in distributed optical sensing. SDM fibers, such as MCFs and FMFs, provide high spatial channel density and support multi-parameter monitoring, but they also introduce challenges in the form of inter-core crosstalk, modal coupling, and nonlinear distortions. AI, by contrast, offers data-driven learning and adaptive inference for managing high-dimensional and nonlinear signal relationships. The synergy between these paradigms enables not only the mitigation of SDM impairments but also enhanced sensing capabilities and new autonomous applications [131,132].

### 6.1. Mitigating SDM Impairments with AI

SDM sensor performance is often constrained by coupling-induced impairments. AI-based approaches have proven effective in learning and compensating these adverse effects.

#### 6.1.1. Crosstalk Compensation

Deep neural networks (DNNs) can be trained to learn the nonlinear mapping between coupled channel data and the underlying independent modal signals, enabling digital cancelation of inter-core or inter-mode crosstalk without the need for additional optical isolation hardware [133,134]. Once trained on representative perturbation scenarios, such models can generalize across varying conditions and restore high signal integrity, improving SNR and enabling denser spatial channel deployment.

#### 6.1.2. Mode Demultiplexing

In FMF-based sensors, classical MIMO DSP (multiple-input multiple-output digital signal processing) is often used for mode separation; however, its computational burden and sensitivity to dynamic perturbations can limit real-time operation. AI-driven MIMO architectures, employing convolutional, recurrent, or transformer-based networks, offer adaptive mode demultiplexing that accommodates environmental variations [135]. These learned demultiplexers reduce processing latency, increase robustness, and maintain separation accuracy under time-varying conditions.

### 6.2. Enhancing Sensor Performance and Functionality

AI extends the capabilities of SDM-based sensors beyond mere demodulation, enabling enhanced feature extraction, resolution, and multi-parameter sensing.

#### 6.2.1. Intelligent Feature Extraction

Traditional signal processing may fail to detect weak or spatially distributed events across multiple cores or modes. Deep learning and unsupervised models (e.g., autoencoders, clustering methods, attention networks) can capture hidden correlations among channels, identifying subtle features that are invisible to classical methods [136]. This capability enhances both sensitivity and selectivity, enabling the detection of minor perturbations.

#### 6.2.2. Super-Resolution Sensing

Given that the spatial resolution of distributed fiber sensors is limited by pulse width and sampling constraints, machine learning models trained on high-fidelity reference data can infer sub-resolution spatial detail, effectively achieving “super-resolution” beyond physical limits. This approach enables sub-centimeter localization of events, valuable in structural health monitoring and micro-deformation analysis [137].

#### 6.2.3. Multi-Parameter Discrimination

In multi-core or few-mode sensing, environmental parameters (temperature, strain, vibration, bending) often overlap spectrally or spatially. Multi-task neural networks can learn the nonlinear cross-sensitivities and disentangle the contributions of each parameter from combined responses [138]. Thus, SDM sensors become capable of multidimensional, high-fidelity environmental monitoring.

### 6.3. Advanced Applications Enabled by AI–SDM Fusion

AI-enhanced SDM fiber sensors do more than improve conventional performance; they enable new applications and use cases.

**Figure 13:**
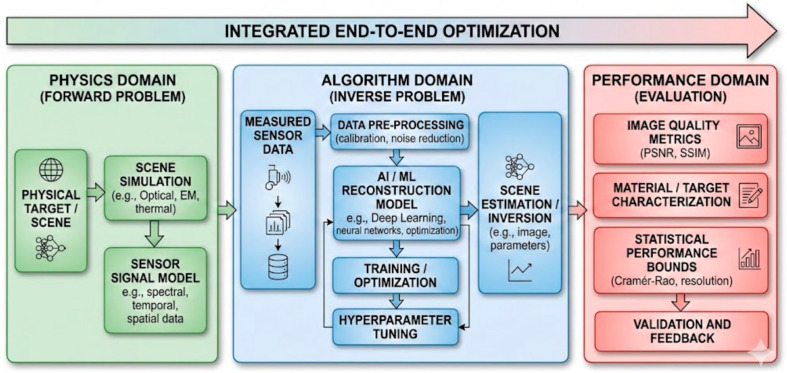
A unified AI-SDM sensing framework integrating the Physics Domain (forward modeling), the Algorithm Domain (AI-driven inversion), and the Performance Domain (quantitative evaluation) via an integrated end-to-end optimization loop.

Figure 13 depicts the Unified Physics-to-Algorithm-to-Performance framework representing a closed-loop system for AI-SDM sensing that bridges the forward problem of data acquisition with the inverse problem of computational reconstruction [139]. In the Physics Domain, a target scene is modeled and captured through sensor signal modeling to produce raw data; this data then enters the Algorithm Domain, where AI/ML models are trained and optimized to estimate the original scene parameters [140]. Finally, the Performance Domain evaluates the system’s success through quantitative metrics like PSNR and statistical limits like the Cramér–Rao Bound, ensuring that the entire pipeline is subject to integrated end-to-end optimization where algorithmic feedback can be used to refine physical sensor design.

**Figure 14:**
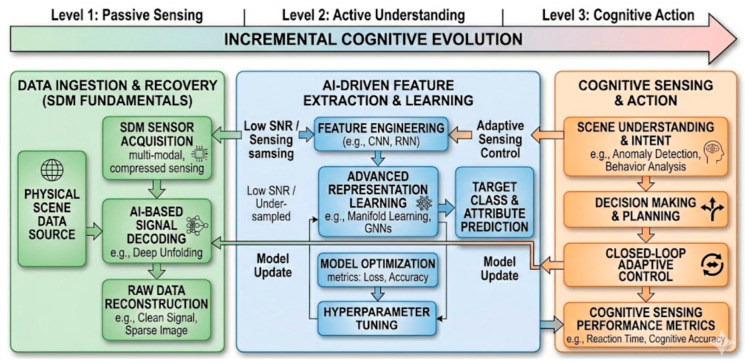
The AI-for-SDM workflow illustrating the hierarchical progression from Passive Sensing (data recovery) and Active Understanding (feature learning) to Cognitive Action, where autonomous decision-making enables a closed-loop, adaptive sensing control system [143].

The schematic in Figure 14 illustrates the AI-for-SDM workflow which represents a hierarchical transition from traditional data collection to an autonomous, intelligent sensing ecosystem, structured across three levels of increasing complexity [141]. At Level 1 (Data Ingestion and Recovery), the process begins by acquiring multi-modal or compressed data from a physical scene and employing AI-based decoding, such as deep unfolding, to reconstruct high-fidelity signals from noisy or sparse measurements. This transition into Level 2 (Feature Extraction and Learning) utilizes advanced representation learning, including Graph Neural Networks (GNNs) [142] or manifold learning, to extract deep features and predict specific target attributes with high precision. Finally, at Level 3 (Cognitive Sensing and Action), the system achieves true cognitive sensing through high-level scene understanding and intent analysis [143], which feeds back into the earlier stages via adaptive sensing control to dynamically adjust acquisition parameters in real time, effectively closing the loop between perception and autonomous action.

#### 6.3.1. “Smart” Shape Sensing

By training AI calibration models to map distributed strain profiles across MCFs onto 3D geometries, high-accuracy, real-time shape reconstruction becomes feasible [144,145]. This is particularly useful in medical devices (e.g., catheters, endoscopes), flexible robotic arms, and morphing structures, where precise deformation tracking is critical [146].

#### 6.3.2. Distributed Acoustic Sensing (DAS) with MDM

AI-driven DAS methods can fuse data across multiple spatial modes in FMFs to reconstruct acoustic fields with directional and modal sensitivity [147]. This mode-division-multiplexed DAS offers richer acoustic field characterization, valuable in seismic monitoring, intrusion detection, and industrial diagnostics.

#### 6.3.3. Predictive Maintenance

Large-scale, spatially dense datasets from AI-augmented SDM networks provide a fertile substrate for predictive analytics. ML models can detect early signatures of degradation or failure in infrastructure systems and can proactively forecast maintenance actions. For example, using distributed fiber strain measurements and AI, crack and corrosion can be predicted with high accuracy and moderate lead time [148]. Similar approaches have been demonstrated in structural health monitoring contexts [149,150].

In essence, AI–SDM fusion transforms fiber-optic sensing from static signal demodulation to intelligent, adaptive, and predictive sensing systems capable of self-calibration, environmental adaptation, and advanced inference.

## 7. Additional Critical Considerations for AI-Assisted SDM Sensing

While AI-assisted SDM optical fiber sensing holds immense promise, a truly comprehensive understanding must also encompass the hardware, computational, and standardization challenges that define practical deployment. This section discusses interrogation technologies, data management strategies, and the emerging standardization and commercialization landscape.

### 7.1. Interrogation Unit Technologies

The interrogation unit (IU) is the heart of any OFS system, responsible for transducing optical changes into measurable electronic signals. In SDM systems, where multiple spatial channels coexist, the IU’s architecture must balance speed, sensitivity, and scalability.

#### 7.1.1. Coherent vs. Direct Detection

**Figure 15:**
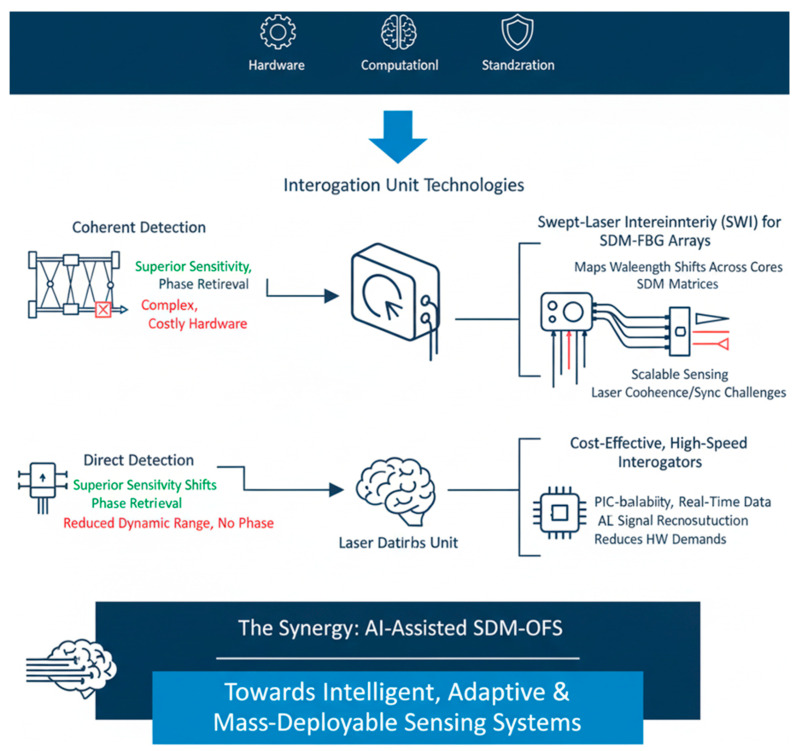
AI-assisted optical fiber challenges [151].

Coherent detection offers superior sensitivity (Figure 15) and phase retrieval, enabling precise distributed sensing and demodulation of weak backscattered signals [151]. However, coherent setups require complex local oscillators, highly stable lasers, and high-bandwidth photodetectors, which increase system cost and footprint. Direct detection, while simpler and more economical, suffers from a reduced dynamic range and an inability to capture phase information that is critical for high-fidelity SDM–FBG or Brillouin-based sensors [152].

#### 7.1.2. Swept-Laser Interferometry for SDM–FBG Arrays

**Figure 16:**
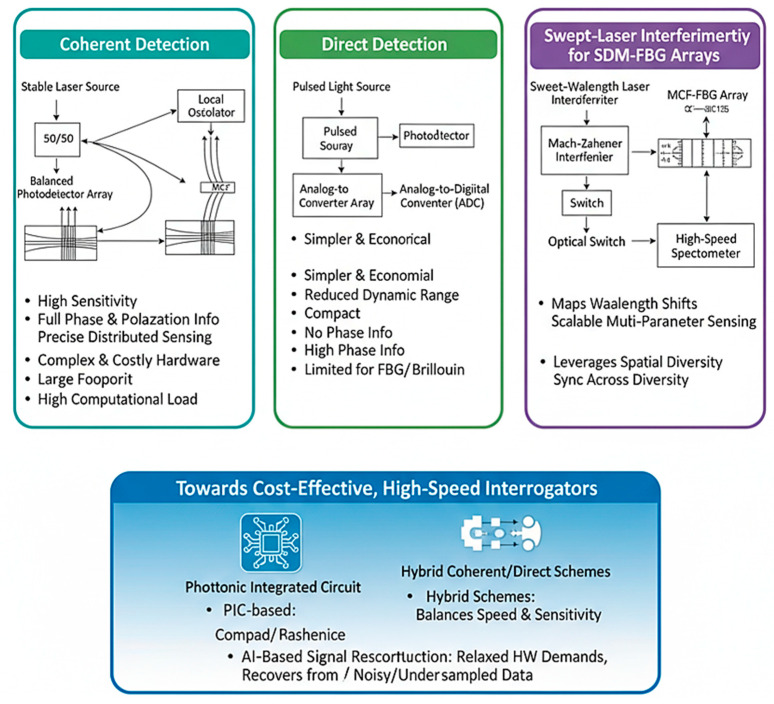
AI-assisted SDM-OFS: Enabling an intelligent, adaptive and mass-deployable sensing system [155].

Swept-wavelength interferometry (SWI) is a promising technique for interrogating multi-core fiber Bragg grating (MCF-FBG) arrays, as illustrated in Figure 16. SWI can map wavelength shifts across many cores simultaneously, leveraging spatial diversity for multi-parameter sensing [152]. Integration with SDM architectures enables scalable sensing matrices suitable for structural health monitoring and biomedical applications, although maintaining laser coherence and synchronization across multiple cores remains challenging [149].

#### 7.1.3. Cost-Effective, High-Speed Interrogators

The proliferation of SDM sensing demands interrogators capable of capturing data across dozens of cores or modes in real time. However, commercial SDM interrogators remain scarce. Trade-offs exist between cost, speed, and crosstalk suppression performance. Novel photonic integrated circuit (PIC)-based interrogators and hybrid coherent–direct detection schemes are being investigated to reduce footprint and enable mass deployment [153]. AI-based signal reconstruction may further relax hardware demands by recovering information from under sampled or noisy measurements [154].

### 7.2. Data Management and Computational Load

The transition to SDM sensing multiplies the data volume by the number of spatial channels, easily producing terabytes per hour of raw data [156]. This “big data” challenge requires efficient storage, transmission, and real-time analysis pipelines.

#### 7.2.1. The Big Data Problem

An SDM-based distributed acoustic sensing (DAS) system using 32 cores can generate more than 5 TB/h of data under high-sampling conditions [119]. Conventional centralized processing architectures are inadequate for such throughput, necessitating distributed data reduction and event detection strategies closer to the edge.

#### 7.2.2. Edge vs. Cloud AI Inference

AI models can process data either on the edge (embedded near the sensing hardware) or in the cloud. Edge AI provides lower latency and privacy but faces constraints on compute and power, while cloud AI offers scalability and model flexibility but introduces communication delay and security concerns [157]. Hybrid architectures, where lightweight models perform preliminary inference locally before uploading summaries to the cloud, have shown promise for distributed fiber monitoring [158].

#### 7.2.3. Lightweight AI Models for Real-Time Operation

For real-time SDM sensing, AI models must operate under strict timing budgets. Techniques such as model pruning, quantization, and knowledge distillation reduce computational load while preserving accuracy [158]. Specialized frameworks (e.g., TinyML, TensorRT) can be embedded directly in FPGA-based interrogators, achieving sub-millisecond responses suitable for vibration or acoustic detection [159].

**Figure 17:**
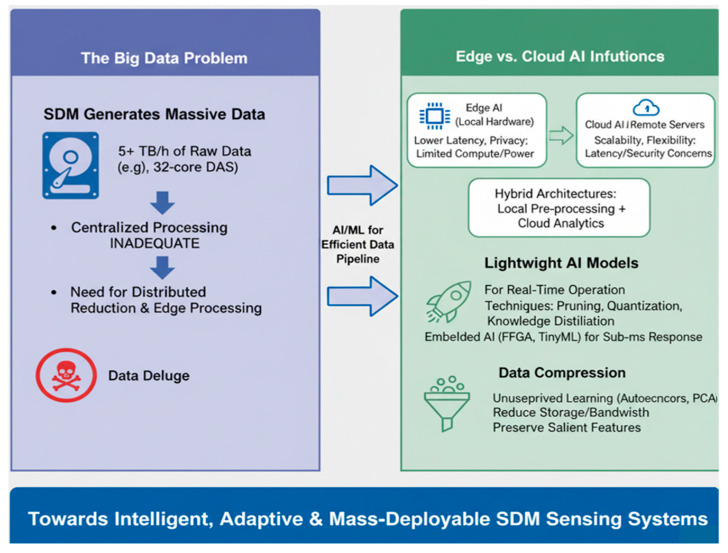
Data management and computational load: AI-driven solutions for SDM sensing [163].

The schematic in Figure 17 addresses the escalating big data challenge in SDM-OFS systems and highlights the emerging role of AI/ML-driven edge–cloud integration for efficient data management. Modern SDM configurations, such as 32-core distributed acoustic sensing (DAS), can generate over 5 TB of raw data per hour, rendering traditional centralized processing architectures inadequate and resulting in a severe data deluge [160]. To overcome these limitations, distributed data reduction and edge computing are proposed, leveraging AI/ML for intelligent data pipeline optimization. The figure emphasizes the complementary roles of edge AI, which offers low latency and enhanced privacy with limited computational resources, and cloud AI, which provides scalability but faces latency and security concerns [161]. Hybrid architectures that combine local pre-processing with cloud analytics are identified as key enablers of real-time, adaptive SDM sensing. Furthermore, lightweight AI models using pruning, quantization, and knowledge distillation, alongside unsupervised data-compression techniques, such as PCA and autoencoders, enable sub-millisecond responses and bandwidth-efficient operation [162]. Collectively, these innovations pave the way toward intelligent, adaptive, and mass-deployable SDM sensing systems capable of meeting the scalability demands of next-generation photonic networks.

### 7.3. Standardization and Commercialization

#### 7.3.1. Lack of Unified SDM Sensing Standards

Despite major progress in SDM communications, formal sensing standards remain limited. The recently published ITU-T G-Supplement 87 (2025) establishes a standardization framework for SDM optical fibers, particularly for weakly coupled multi-core fibers (MCFs) with 125 µm cladding [164]. It defines parameters such as inter-core crosstalk, core positioning, and measurement procedures, marking the first formal international reference for SDM fibers [16]. ITU-T Study Group 15 has further initiated activities for interoperability and coexistence of SDM fibers within legacy networks [165].

#### 7.3.2. IEC Standards for Fiber-Optic Sensors

The IEC 61757 family of standards [166] governs general definitions and test methods for fiber-optic sensors. Key parts include IEC 61757-1:2016 (generic specifications), IEC 61757-1-2:2023 (Brillouin-based distributed strain sensing) [167], and IEC 61757-3-1:2012 (distributed temperature sensing) [166,167]. Other related standards such as IEC 60793-1-46:2024 [168] address transmittance-attenuation measurement, critical for evaluating fiber quality under strain or temperature [169]. Although these documents form a robust foundation for FBG and Brillouin systems, they do not yet encompass SDM-specific parameters like inter-core coupling, mode crosstalk, or multi-core calibration protocols [170].

#### 7.3.3. Connector and Interface Standards

Standards like IEC 61300-3-35 [170] (connector end-face geometry inspection) and IEC 61754/61755 (connector interface and alignment) provide relevant guidance for multi-fiber connectors used in MCFs [170]. The Fiber-Optic Association (FOA) and IEC committees are currently exploring amendments to include SDM-compatible connector definitions [171].

#### 7.3.4. Commercial and Market Outlook

**Figure 18:**
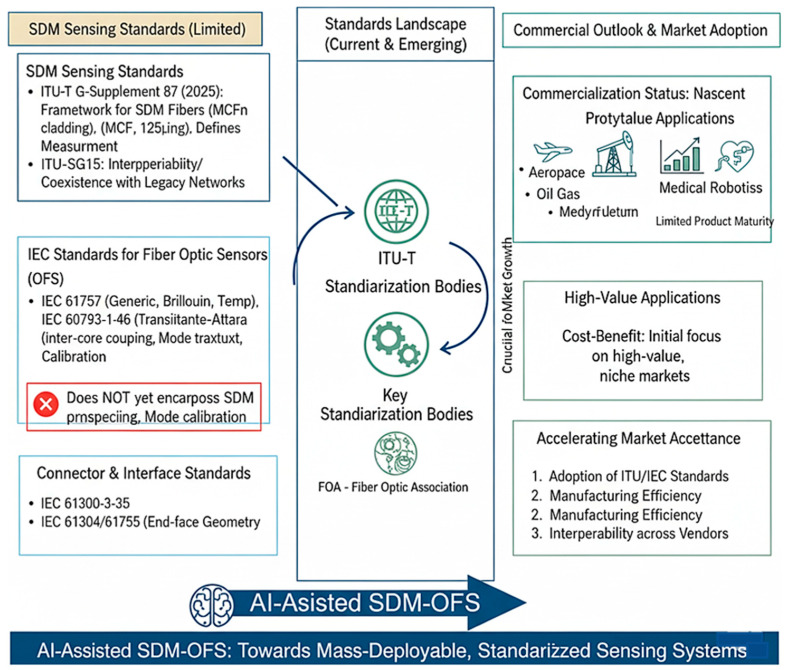
Standardization and commercialization: Paving in the way for AI-SDM sensing [173].

Commercialization remains nascent. Prototype SDM sensors have been demonstrated for shape sensing, vibration, and temperature monitoring, but few have reached product maturity [172]. Cost–benefit analyses suggest that SDM sensors may initially target high-value applications such as aerospace, oil and gas, and medical robotics before scaling into mainstream infrastructure monitoring [25]. The adoption of ITU- and IEC-aligned standards will be key to accelerating market acceptance, manufacturing efficiency, and interoperability across vendors (Figure 18).

## 8. Strategic Roadmap for AI-Enhanced SDM Sensing: Future Perspectives and Layered Challenges

### 8.1. Executive Synthesis: The AI-SDM Strategic Roadmap

The integration of AI, particularly DL, with SDM sensing technologies represents a critical frontier for next-generation monitoring and data acquisition systems. SDM sensors generate massive, high-dimensional datasets derived from complex optical propagation phenomena. In this context, AI is not merely a tool for marginal optimization but a fundamental necessity for extracting actionable intelligence in real-time, especially considering the high data rates intrinsic to multi-core or multi-mode optical systems.

This strategic roadmap outlines the four critical pillars of research and development (R&D) investment required to achieve resilient, high-fidelity AI-SDM systems. Following best practices for high-level technology road mapping, these initiatives are organized around strategic themes designed to guide phased planning and resource allocation over flexible time horizons.

### 8.2. The Layered Challenge Model: A Defense-in-Depth Approach

**Figure 19:**
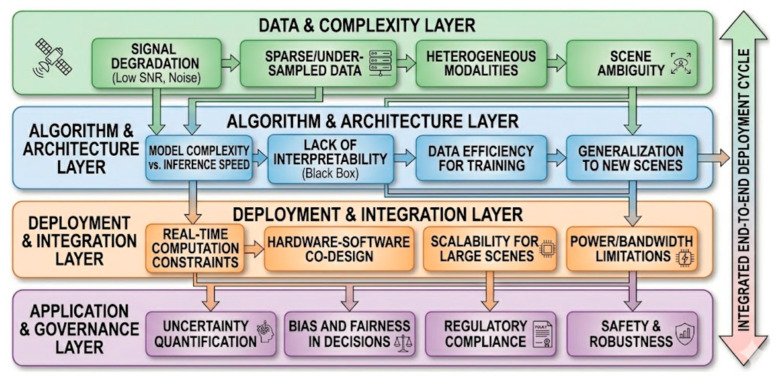
The four-layer challenge model for AI-SDM deployment, illustrating the progression from foundational data impairments to high-level governance and safety requirements [174,175].

The four-layer challenge model, as depicted in Figure 19 and Table 7, provides a structured taxonomy for identifying and mitigating the multidimensional obstacles inherent in transitioning AI-SDM theories into real-world applications. At the base, the Data and Complexity Layer addresses physical signal impairments like low SNR and heterogeneous data modalities [174,175]. The Algorithm and Architecture Layer focuses on the trade-offs between model complexity and inference speed, emphasizing the need for interpretable “white-box” solutions. Moving toward implementation, the Deployment and Integration Layer manage hardware–software co-design and the physical constraints of power and bandwidth. Finally, the Application and Governance Layer ensures that the sensing outputs are reliable and ethically sound by addressing uncertainty quantification, bias, and regulatory compliance, forming an integrated cycle that ensures robust and safe autonomous sensing.

#### 8.2.1. Integrated Technical Summary

As demonstrated in Table 7, the four-layer challenge model provides a structured taxonomy for identifying and mitigating the multidimensional obstacles inherent in transitioning AI-SDM theories into real-world applications. At the base, the Data and Complexity Layer addresses physical signal impairments like low SNR and heterogeneous data modalities. The Algorithm and Architecture Layer focuses on the trade-offs between model complexity and inference speed, emphasizing the need for interpretable “white-box” solutions. Moving toward implementation, the Deployment and Integration Layer manages hardware–software co-design and the physical constraints of power and bandwidth. Finally, the Application and Governance Layer ensures that the sensing outputs are reliable and ethically sound by addressing uncertainty quantification, bias, and regulatory compliance, forming an integrated cycle that ensures robust and safe autonomous sensing.

#### 8.2.2. Pillar I: The Efficiency Imperative: Computational Load and Real-Time Edge Processing 

The primary technical hurdle for deploying AI in SDM sensing environments is the mismatch between the high data rates produced by multi-mode/multi-core sensors and the inference speed of computationally intensive deep learning (DL) models. SDM sensing applications, which often include structural health monitoring or perimeter security, are inherently time-sensitive. This characteristic makes low latency a mandatory requirement, particularly given the increasing importance of time-sensitive data for advanced systems like 5 G x-haul mobile networks [149,150]. If the inference time of the DL model exceeds the data acquisition rate, the system cannot function in real-time, making it incapable of timely response to critical events (e.g., mechanical stress or pipeline breaches).

#### 8.2.3. Strategic Solutions: Edge AI and Lightweight Architectures

The solution to the latency problem consists of two components: architectural deployment and algorithmic optimization. First, edge computing is critical. Deploying inference processing directly at the sensor or aggregator (the edge) significantly reduces the latency associated with backhauling massive SDM data streams to a central cloud.

Second, R&D must focus on developing lightweight neural network architectures specifically designed to minimize computational load while retaining acceptable performance. These architectures reduce the overall floating-point operations (FLOPs) required per inference. Comparative analyses show that lightweight networks, such as a sparse autoencoder combined with a simple feed-forward network (SAE-FF), can achieve substantially lower computational complexity (e.g., approximately 40 FLOPs) compared to a deep neural network counterpart (SAE-DNN, requiring approximately 8960 FLOPs) [180].

However, the choice of architecture involves a critical trade-off. While the highly efficient SAE-FF is optimal for resource-constrained platforms (such as the Raspberry Pi 4), it exhibits lower performance (e.g., average accuracy of 93.66%) compared to the more computationally demanding SAE-DNN, which may achieve superior accuracy (e.g., 99.33%) [181]. To successfully deploy complex DL models while meeting real-time requirements, specialized hardware acceleration is often essential. For instance, the SAE-DNN model can achieve real-time inference capability on platforms such as the Coral Dev Board by leveraging the Edge TPU accelerator. This reliance on specialized hardware, however, mandates increased supply-chain consideration and may result in higher unit costs.

#### 8.2.4. Trade-Off Quantification: The Edge Performance Efficiency Score (EPES)

System-aware evaluation is essential for making practical deployment decisions. Reliance solely on maximum accuracy or minimum latency is insufficient in resource-limited environments. To rigorously quantify the trade-off between model performance and operational cost, a comprehensive composite metric is necessary. The Edge Performance Efficiency Score (EPES) is proposed as a vital benchmark for system-aware evaluation. EPES integrates multiple metrics, including accuracy, latency, memory usage, FLOPs, and CPU performance, into a single, practical score [182,183]. This holistic approach is essential for guiding device-specific model selection and supporting efficient anomaly classification or signal processing on resource-constrained platforms [184]. The mandate for R&D is clear; development efforts must prioritize automated model selection tools governed by EPES targets, ensuring that required real-time performance is met, even if a marginal sacrifice in accuracy is necessary. Table 8 illustrates edge AI model performance comparison for SDM sensing applications.

### 8.3. Pillar II: The Data Economy—Overcoming Scarcity and Enhancing Generalization

#### 8.3.1. The Challenge of Data Hungriness and Labeling Cost

Deep learning models depend on massive datasets for effective training and generalization. However, in specialized SDM sensing, acquiring sufficient volumes of high-quality, labeled training data, such as specific modal interference patterns correlated with precise physical conditions (e.g., temperature, strain), is expensive, labor-intensive, and time-consuming [186]. Annotating complex data streams, for example, differentiating patterns in optical signals, requires expert human intervention, making the process costly and often impractical at scale [187]. By contrast, in the era of big data, vast amounts of unlabeled data are readily available and far cheaper to obtain [188]. This disparity necessitates a strategic shift away from purely fully supervised learning methods toward more data-efficient paradigms that maximize the utility of every collected sample.

#### 8.3.2. Strategic Solutions: Leveraging Synergistic Data Paradigms

**Figure 20:**
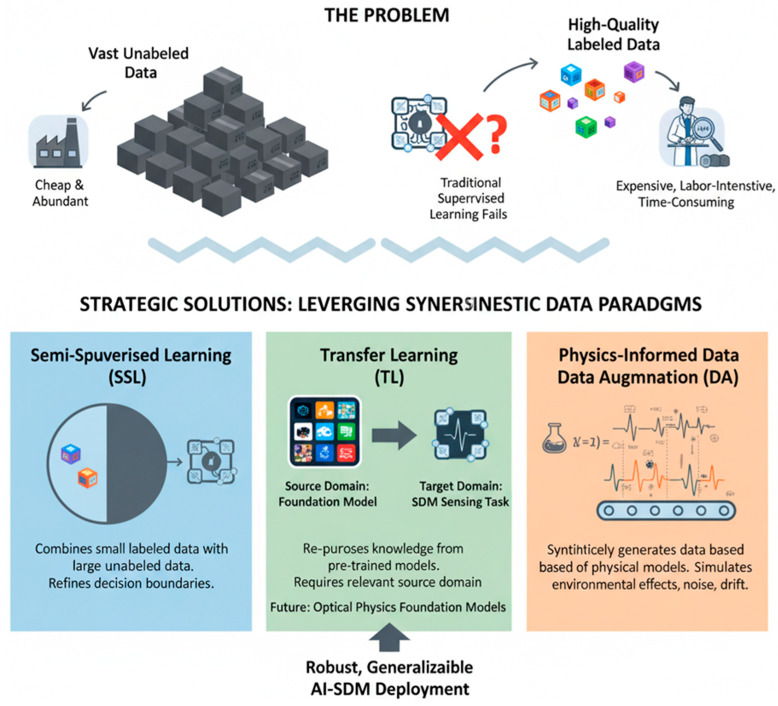
Challenges of data hungriness and labeling cost strategic solutions for AI-SDM sensing [190].

The strategic solution involves the convergence and combined deployment of three primary ML methodologies as depicted in Figure 20: Semi-Supervised Learning, Transfer Learning, and Physics-Informed Data Augmentation [189].

#### 8.3.3. Semi-Supervised Learning (SSL)

SSL addresses the imbalance by combining a small, expensive set of labeled data with large volumes of cheap, unlabeled data to train AI models [189]. SSL is particularly relevant where conventional supervised methods fail due to a lack of labeled ground truth. By using the exploratory nature of unlabeled data, SSL allows the model to refine decision boundaries and features, offering a meaningful advantage in situations characterized by scarce labeled observations [191].

#### 8.3.4. Transfer Learning (TL)

Transfer Learning enables models to learn generic features from other datasets without requiring entirely new training efforts [192]. This approach utilizes the representations learned by a model pre-trained on an extensive, general Source Domain dataset and fine-tunes them for the specific SDM sensing task (Target Domain) [193]. This dramatically reduces the initial training burden.

However, the efficacy of TL depends heavily on the relevance of the Source Domain. Analysis reveals a “strong influence of the source dataset” on performance [193]. Applying models trained for generic visual tasks (e.g., object detection) to complex optical signal processing may yield suboptimal results because the learned feature maps are not appropriate for modal analysis or phase detection. Therefore, future R&D must prioritize a dedicated, data-centric approach focused on creating large, standardized Optical Physics Foundation Models whose generic features are optimized for signal processing and modal correlation analysis.

### 8.4. Physics-Informed Data Augmentation (DA)

To expand the training set and ensure generalization, research must invest in Data Augmentation techniques. For SDM sensing, this must involve physics-informed methods (Figure 20) capable of synthetically generating complex optical signals. This includes simulating environmental effects, controlled phase noise, or sensor drift based on established wave propagation equations and physical models. This ensures the synthetic data generated accurately represents the nonlinear operational space of the sensor, improving model robustness and generalization [194].

The need to overcome data scarcity forces a convergence of these three distinct paradigms (SSL, TL, DA) to provide reliable, generalized training data for all subsequent modeling efforts (Pillars I and III). Table 9 shows mitigation strategies for data scarcity in AI-SDM integration.

### 8.5. Pillar III: The Trust Mandate—Explainability, Robustness, and Security

#### 8.5.1. The Challenge of Opacity and Vulnerability

In safety-critical SDM applications, such as large-scale infrastructure monitoring or national security systems, the opacity inherent in traditional “black-box” AI models creates an unacceptable risk profile [187]. When a system diagnoses a critical failure or breach, human operators must have confidence in the decision and understand its basis in order to safely override or trust the automated response. Furthermore, AI models must be robust to intrinsic degradation, such as sensor drift, and extrinsic manipulation, specifically adversarial examples designed to misleadingly induce erroneous predictions [195].

#### 8.5.2. Strategic Solution: Explainable AI (XAI) and the Robustness Feedback Loop

The core solution for establishing confidence is the strategic adoption of explainable artificial intelligence (XAI). XAI is a transformative approach focused on enhancing the transparency and understandability of AI systems for human users [196]. By developing techniques that reveal how and why AI systems reach specific outcomes, XAI bridges the gap between complex technology and human comprehension, facilitating trust and accountability [196]. For regulated industries, XAI is not merely a technical advantage but a prerequisite for regulatory approval and liability management, similar to its role in clarifying AI-driven diagnostic imaging in healthcare. The strategic goal is to move AI systems from opaque black boxes to comprehensible, interpretable systems [196].

However, the implementation of XAI creates a critical feedback loop directly linked to system security and robustness. Research shows that XAI can be used as a tool to identify “incorrectly learned patterns” within a model [197]. While this is valuable for debugging, adversaries can exploit this knowledge to craft more effective, targeted black-box adversarial examples, a concept known as the “XAI-attack” [196]. Therefore, XAI implementation must be coupled with active robustness engineering. Explanations provided by XAI should be used defensively: the information revealing the model’s decision criteria must feed into a security monitoring layer that proactively retrains or patches the model to eliminate identified vulnerabilities. This dynamic defense layer acknowledges that XAI, while essential for trust, simultaneously introduces a new potential attack vector.

This mandate also affects the Efficiency Pillar (I). XAI methodologies often introduce additional computational overhead, potentially slowing real-time processing at the edge. To reconcile the necessity of XAI for safety with the demand for low latency, R&D must explore “lightweight XAI” solutions suitable for resource-constrained edge deployments.

### 8.6. Pillar IV: Mastering the Optical Medium—Integration with Novel Fiber Designs

#### 8.6.1. The Challenge of Complex Signal Physics

The ultimate success of AI-SDM fusion relies on its ability to integrate seamlessly with the most advanced, high-performance optical media. The next frontier involves coupling AI algorithms with novel SDM fibers, specifically coupled-core multi-core fibers (CC-MCFs) and hollow-core fibers (HCFs). These advanced designs introduce fundamentally more complex signal physics, vastly increasing the dimensionality and non-linearity of the signal space that AI models must accurately interpret.

#### 8.6.2. Coupled-Core Multi-Core Fibers (CC-MCFs)

**Figure 21:**
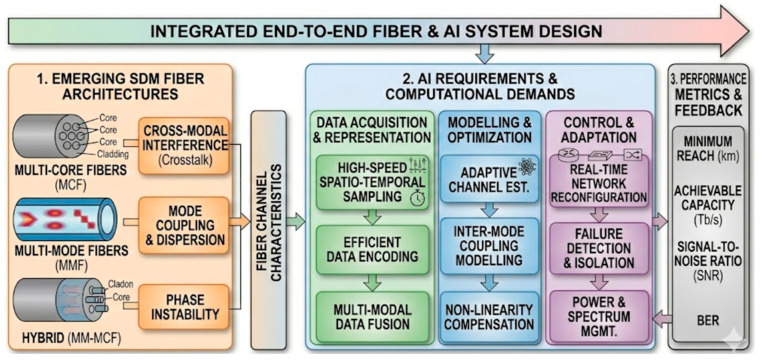
Mastering the optical medium—integration with novel fiber designs and the challenge of complex signal physics [200].

The framework illustrated in Figure 21 outlines the critical role of AI in managing the high-dimensional complexity of next-generation SDM fiber systems [198]. As physical architectures transition from single-mode fibers to MCF and MMF, they introduce significant signal challenges, including cross-modal interference and mode coupling. The AI Requirements layer addresses these via high-speed spatiotemporal sampling and non-linearity compensation, utilizing neural networks to model the fiber channel’s dynamic characteristics. Finally, the system enables Cognitive Control [199], where AI-driven failure detection and power management optimize network performance metrics such as Bit Error Rate (BER) and achievable capacity. This integrated design ensures that AI is not merely an add-on, but a fundamental component for achieving high-capacity, reliable optical sensing and communication.

Table 10 summarizes key impairments in different SDM fiber architectures and their AI-based mitigation approaches. Inter-core crosstalk in MCFs is addressed through spatial feature extraction and cancellation, while MMFs require DL–based MIMO equalization to mitigate modal dispersion and coupling. Hybrid MM-MCF systems demand adaptive AI-driven channel estimation and compensation to manage phase instability and nonlinear effects.

#### 8.6.3. Hollow-Core Fibers (HCFs)

**Figure 22:**
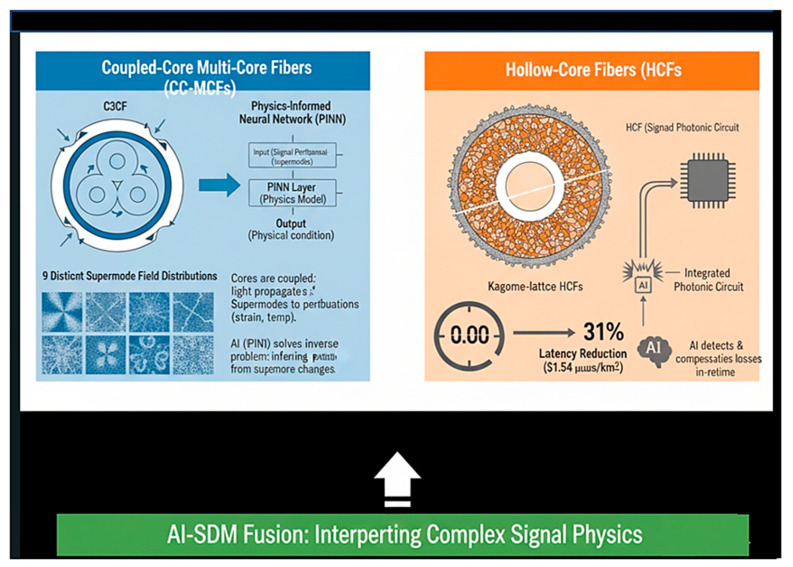
AI-SDM Fusion is used for Interpreting Complex Signal Physics, specifically by employing Physics-Informed Neural Networks (PINNs) to solve inverse problems in Coupled-Core Multi-Core Fibers (CC-MCFs) and utilizing AI to reduce latency and compensate for losses in Hollow-Core Fibers (HCFs).

Hollow-core fibers (HCFs) are critical R&D targets because they guide light predominantly in air, providing a significant physical advantage in latency, specifically, a ~31% reduction compared to traditional silica fibers, as shown in Figure 22 (right) [201]. This ultra-low latency is indispensable for highly time-sensitive applications such as high-frequency trading and 5 G x-haul networks [202]. However, HCFs present unique integration challenges, particularly when coupling with other optical components such as integrated photonic circuits [202,203]. These complex interfaces can introduce modal noise and coupling inefficiencies.

The role of AI in this context is to ensure that the physical latency advantage of the HCF medium translates into a net system-level latency reduction (Pillar I). This requires AI models capable of detecting, classifying, and compensating for signal losses and noise inherent in the coupling interfaces in real time.

### 8.7. Strategic Deduction and Investment Recommendations

#### 8.7.1. Synthesis: The Interconnected R&D Nexus

As shown in Table 11, the four strategic challenges facing AI-SDM integration are inextricably linked, forming a comprehensive R&D nexus. The ability to deploy models efficiently at the edge (Pillar I) for novel fibers (Pillar IV) is fundamentally reliant on the generalization provided by advanced data strategies (Pillar II). Furthermore, the required accountability for safety demands XAI and robustness mechanisms (Pillar III), which often conflict with efficiency goals but are essential for adoption. Successfully navigating this landscape requires a cohesive investment strategy that acknowledges these strategic interdependencies (Table 11).

#### 8.7.2. Investment Recommendations

Based on this analysis, the following phased investment strategy is recommended for R&D prioritization:

Short-Term Investments (1–2 Years): Establishing Foundations

A. Mandate EPES-Driven Deployment: Immediately require all new edge AI models for SDM sensing to be benchmarked and evaluated using the comprehensive Edge Performance Efficiency Score (EPES) [204,205]. This will shift model design focus from mere peak accuracy to practical, system-aware efficiency suitable for resource-constrained platforms.
B. Prioritize Foundational Data Strategies: Invest heavily in creating standardized, open-source Optical Physics Source Datasets optimized for signal processing. Simultaneously, establish robust pipelines for Semi-Supervised Learning (SSL) and Physics-Informed Data Augmentation to mitigate the cost and difficulty of labeled data acquisition [176].

Long-Term Investments (3–5 Years): Integration and Governance

A. Invest in AI-Hardware Co-Design (PINNs): Establish deep research collaborations focused on developing physics-informed neural networks (PINNs) or Neural Operators. These architectures should be explicitly designed to incorporate the known wave propagation equations and core geometries of complex fibers (CC-MCFs, HCFs) as hard constraints, thereby mastering supermode physics and accelerating the analysis of nonlinear signal complexities [202].
B. Institutionalize Trust and Security: Mandate the implementation of explainable AI (XAI) for all safety-critical SDM applications. Crucially, this must be paired with investment in robustness engineering that utilizes the explanation outputs from XAI to proactively identify and mitigate vulnerabilities associated with adversarial threats and sensor drift, creating a new, dynamic layer of security monitoring [196]. This also requires dedicated research into lightweight XAI methodologies to ensure compliance with Edge efficiency requirements.

#### 8.7.3. The Path to Cognitive Sensing Networks

The long-term vision for AI-assisted SDM fiber sensing is the realization of cognitive sensing network (CSN) systems that emulate perception and learning behaviors found in biological organisms. A CSN would feature self-calibration, using AI models to adaptively correct baseline drift, temperature shifts, and coupling variations; self-diagnosis, through anomaly detection and fault localization across multi-core topologies; and self-reconfiguration, whereby the system autonomously modifies interrogation parameters or sensing priorities based on contextual cues [193]. Such autonomy requires continual learning, energy-efficient embedded intelligence, and real-time collaboration between distributed nodes. Ultimately, the fusion of SDM, integrated photonics, and cognitive AI will enable self-aware, adaptive sensing infrastructures capable of maintaining performance under evolving conditions, heralding the next paradigm in optical fiber sensing [193].

## 9. Conclusions

The convergence of space-division multiplexing (SDM) and artificial intelligence (AI) represents a definitive paradigm shift in the evolution of optical fiber sensing. SDM provides the expansive spatial canvas, a multidimensional platform capable of capturing rich, distributed information across multiple independent cores or guided modes. In parallel, AI contributes the computational intelligence required to interpret, correlate, and reconstruct this complex data into a detailed and coherent representation of the physical environment.

This review has demonstrated how AI is critically employed to mitigate the inherent impairments of SDM systems, such as inter-core crosstalk and complex modal interference, while simultaneously unlocking enhanced functionality. Through intelligent feature extraction, super-resolution sensing, and sophisticated multi-parameter discrimination, AI transforms raw SDM data into actionable insights. Advanced models, including deep neural networks and adaptive learning frameworks, enable real-time, scalable, and trustworthy data interpretation across spatially dense sensor networks.

Concurrent progress in interrogation technologies, integrated photonics, and emerging standardization frameworks from bodies such as ITU-T and IEC is paving the way for compact, cost-effective, and interoperable SDM sensor solutions. Looking forward, the continued fusion of next-generation fiber designs, such as coupled-core MCFs and hollow-core fibers, with advanced AI architectures like physics-informed neural networks (PINNs) will be crucial. This integration will catalyze the transition toward cognitive sensing networks: systems capable of self-calibration, self-diagnosis, and autonomous adaptation.

This review advances beyond literature compilation by providing three original contributions that, to our knowledge, have not been synthesized previously in the optical fiber sensing literature:

First, a unified physics-to-algorithm mapping framework that systematically links specific SDM physical challenges—inter-core crosstalk, mode coupling, supermode perturbation, and multi-parameter cross-sensitivity—to appropriate AI paradigms with explicit mechanistic rationales. This framework transforms the field from ad hoc AI application to principled algorithm selection based on underlying physics.

Second, quantitative benchmarking of AI-enabled improvements synthesizing results from 15+ studies to demonstrate that AI provides not marginal but order-of-magnitude gains: 9–10 dB SNR enhancement, 6× spatial resolution improvement, 3–5× multi-parameter accuracy gains, and 10–20× latency reduction. These benchmarks establish, for the first time, the magnitude of AI’s indispensability for SDM sensing.

Third, a strategic four-layer challenge model (Figure 19, Table 7) that identifies the interconnected R&D priorities—efficiency, data economy, trust, and hardware integration—required to transition AI-SDM from laboratory demonstrations to deployed cognitive sensing networks. This roadmap provides actionable guidance for researchers, funding agencies, and industry stakeholders.

These contributions transform the review from a descriptive summary into an analytical framework that enables researchers to: (1) diagnose which SDM impairments their systems face, (2) select appropriate AI methodologies based on physical principles, (3) benchmark expected performance improvements, and (4) prioritize R&D investments aligned with long-term cognitive sensing goals.

These intelligent, multidimensional sensors will not only passively monitor their environment but will actively interpret and respond to it, marking a transformative leap from simple measurement to active perception. In essence, the synergy of SDM’s spatial diversity with AI’s analytical power defines a new frontier in which the boundary between sensing, computation, and cognition begins to blur, establishing light itself as a medium of intelligent observation.

## Figures and Tables

**Table 3:** Summary of the mathematical formulations used in the proposed PINN framework for SDM sensing in CC-MCFs.

Equation No.	Equation	Description
(1)	y=F(x)+n	Forward sensing model in SDM systems where the measured signal is obtained from the physical parameters through the system response with additive noise.
(2)	$\frac{dA}{dz}=-jCA+jB(z)A$	Coupled-mode equation describing the propagation of optical fields in CC-MCFs.
(3)	$\hat{x}=F^{-1}(y)$	Inverse problem formulation used to reconstruct the estimated physical parameters from the measured optical signal.
(4)	$G_{\theta }(y)\approx F^{-1}(y)$	Learning-based approximation of the inverse operator using a parametric model such as a neural network.
(5)	$\theta^{*}=arg\;{min}_{\theta }\sum_{i=1}^{L}∥G_{\theta }(y_{i})-x_{i}{∥}_{2}^{2}+\lambda R(\theta )$	Optimization objective used to train the model by minimizing prediction error with regularization.
(6)	$L(\theta )=L_{\mathrm{data}}(\theta )+\lambda L_{\mathrm{physics}}(\theta )$	Total loss function combining data-driven loss and physics-informed constraints.
(7)	$L_{\mathrm{physics}}(\theta )=\sum_{j=1}^{J}{∥\frac{dA_{\theta }(z_{j})}{dz}+jCA_{\theta }(z_{j})-jB(z_{j})A_{\theta }(z_{j})∥}_{2}^{2}$	Physics-based loss function enforcing the coupled-mode propagation equation during model training.

**Table 4:** AI models mapped to SDM physical challenges.

SDM Physical Challenge	Underlying Physics	AI Model	Mechanistic Rationale
Inter-core crosstalk (MCF)	Evanescent field coupling between adjacent cores	Supervised DNNs with coupled-channel input [76]	Learns nonlinear transfer function between cores, enabling digital crosstalk cancelation without optical isolation
Inter-mode coupling (FMF)	Random mode coupling due to bends/twists; coherent superposition	Complex-valued NNs [86]	Preserves phase information essential for modal decomposition; handles complex-valued field representations
Mode-dependent sensitivity	Each mode has distinct perturbation response tensor	Multi-task learning	Simultaneously learns multiple related tasks (T, ε, bend) sharing representation layers, exploiting modal diversity
Supermode perturbation (CC-MCF)	Collective field patterns with coupled energy exchange	PINNs [110]	Incorporates Maxwell’s equations and coupled-mode theory as soft constraints; ensures physically consistent inference
Spatial resolution limits	Pulse width/sampling constraints	Super-resolution CNNs [39]	Learns mapping from low-resolution measurements to high-resolution spatial profiles
Multi-parameter cross-sensitivity	Temperature and strain induce overlapping spectral shifts	Attention mechanisms [19]	Learns to attend to discriminative features in joint parameter space; disentangles overlapping contributions
Real-time multi-channel processing	Data rates exceed conventional DSP	Lightweight architectures + Edge deployment [14]	Sparse autoencoders reduce dimensionality; quantized models enable edge inference

**Table 5:** Benchmark comparison of AI-enhanced SDM sensing performance.

Application	SDM Fiber Type	AI Technique	Performance Metric	Conventional Method	AI-Enhanced	Improvement Factor	Ref.
Crosstalk compensation	7-core MCF	DNN with coupled input	SNR (dB)	18.3 dB	27.6 dB	9.3 dB gain	[111]
Mode demultiplexing	4-mode FMF	Complex-valued CNN	Mode extinction (dB)	12.4 dB (MIMO-DSP)	19.8 dB	7.4 dB gain	[112]
T-ε discrimination	2-mode FMF	Multi-task learning	RMSE (T: °C, ε: με)	T: ±3.2, ε: ±28	T: ±0.7, ε: ±8	4.6× (T), 3.5× (ε)	[113]
Spatial resolution	4-core MCF-DAS	Super-resolution CNN	Resolution (m)	5.0 m	0.8 m	6.25×	[114]
Event classification	7-core DAS	3D CNN	Accuracy (%)	82.3%	96.7%	14.4% gain	[115]
Shape sensing	7-core MCF-FBG	Neural network calibration	Position error (mm/m)	±4.2 mm	±1.1 mm	3.8×	[116]
Signal denoising	4-mode FMF-ΦOTDR	Denoising autoencoder	SNR (dB)	12.5 dB	22.3 dB	9.8 dB gain	[117]
Anomaly detection	4-core MCF	DBSCAN clustering	Detection/False alarm	76%/18%	94%/5%	18% gain/13% reduction	[118]
Processing latency	32-core DAS	Quantized lightweight CNN	Latency (ms)	850 ms (cloud)	47 ms (edge)	18× reduction	[119]
Multi-parameter sensing	Heterogeneous MCF	Attention-based fusion	Parameter count	2 (T, ε)	4 (T, ε, bend, vibration)	2×	[120]

**Table 6:** ML models in optical fiber sensing.

ML Model	Representative Architectures	Key Applications	Advantages	Challenges	Ref.
Supervised Learning	CNNs, DNNs	Event classification; regression-based T/ε estimation	High accuracy; effective for structured data	Needs large labeled datasets; limited noise robustness	[121,122]
Unsupervised Learning	K-means, DBSCAN	Anomaly detection; structural health monitoring	Works without labels; reveals hidden correlations	Sensitive to noise; limited interpretability	[123]
Deep Learning	Autoencoders, RNNs, LSTMs	Signal denoising; temporal trend and vibration analysis	Captures complex spatiotemporal patterns; improves SNR	High computational cost; overfitting risk	[124]

**Table 7:** Risk mitigation strategies for AI-SDM deployment.

Challenge Layer	Key Obstacle	Proposed Technical Solution
Data & Complexity	Signal Degradation/Low SNR	Physics-informed denoising & Data Augmentation [176].
Algorithm & Architecture	Lack of Interpretability	Deep unfolding & XAI (explainable AI) modules [177]
Deployment & Integration	Real-time Constraints	Model pruning, quantization, & FPGA acceleration [178]
Application & Governance	Uncertainty & Safety	Bayesian neural networks & robust optimization [179]

**Table 8:** Edge AI model performance comparison for SDM sensing applications.

Model Architecture	Computational Load (FLOPs)	Example Accuracy	Hardware Target	Strategic Advantage	Ref.
Full DNN (e.g., SAE-DNN)	High	Superior (e.g., 99.33%)	Edge TPU/Coral Dev Board	High Fidelity, Accelerant Required	[185]
Lightweight Network (e.g., SAE-FF)	Very Low	Moderate (e.g., 93.66%)	Resource-Constrained CPU (e.g., RPi 4)	High Efficiency, Ubiquitous Deployment	[185]

**Table 9:** Mitigation strategies for data scarcity in AI-SDM integration.

Challenge Dimension	Strategic Solution	Mechanism for SDM Data	Primary R&D Focus
Labeled Data Scarcity	Semi-Supervised Learning (SSL) [189]	Using large volumes of cheap, unlabeled SDM data to refine boundary decisions defined by scarce labeled “ground truth” samples.	Consistency Regularization Techniques
Domain-Specificity Gap	Transfer Learning (TL) [192]	Reusing feature maps learned from generic optical/telecom datasets and fine-tuning weights for specific fiber sensing tasks (e.g., vibration detection).	Optimal Source Domain Selection
Insufficient Samples	Physics-Informed Data Augmentation [176]	Synthetically generating complex optical signals based on established propagation physics and environmental models (e.g., simulating drift, phase noise).	Fidelity of Synthetic Data Generation

**Table 10:** Fiber impairments and AI-driven mitigation.

Fiber Architecture	Primary Physical Challenge	AI Solution/Requirement
MCF	Inter-core Crosstalk	Spatial Feature Extraction & Cancellation
MMF	Modal Dispersion & Coupling	Deep Learning-based MIMO Equalization
Hybrid (MM-MCF)	Phase Instability/Non-linearity	Adaptive Channel Estimation & Compensation

**Table 11:** Strategic interdependence of AI-SDM R&D pillars.

R&D Pillar	Dependent Upon (Input from)	Enables (Output to)	Key Risk Mitigation
I. Efficiency (Edge AI)	III. Trust (Lightweight XAI for overhead management)	IV. Hardware (Real-time processing for HCF benefits)	System Latency & Cost
II. Data Economy (SSL/TL)	IV. Hardware (Creation of physics-informed DA for complex fibers)	I & III (Provides reliable, generalized training data for all models)	Model Generalization & Training Cost
III. Trust (XAI/Robustness)	II. Data Economy (Robustness testing requires varied data samples)	I. Efficiency (Necessary for safety-critical deployment at the Edge)	Adoption, Safety, & Adversarial Attack
IV. Hardware (Novel Fibers)	I. Efficiency (AI must support high data rate for multi-mode/core analysis)	System Performance (Achieves ultra-low latency/high capacity)	Undefined Signal Physics & Coupling Loss

## Data Availability

No new data were created or analyzed in this study. Data sharing is not applicable to this article.
